# Reducing drug-use harms among higher education students: MyUSE contextual-behaviour change digital intervention development using the Behaviour Change Wheel

**DOI:** 10.1186/s12954-021-00491-7

**Published:** 2021-05-20

**Authors:** Vasilis S. Vasiliou, Samantha Dockray, Samantha Dick, Martin P. Davoren, Ciara Heavin, Conor Linehan, Michael Byrne

**Affiliations:** 1 School of Applied Psychology, University College Cork, N Mall, Kilbarry Enterprise Centre, Cork Enterprise Centre, Cork, Ireland; 2 School of Public Health, University College Cork, Fourth Floor, Western Gateway Building, Cork, Ireland; 3 Cork Sexual Health Centre, 16 Peter’s Street, Centre, Cork, Ireland; 4 Cork University Business School, University College Cork, West Wing, Main Quadrangle, Cork, Ireland; 5 Student Health Department, University College Cork, Ardpatrick College Road, Cork, Ireland

**Keywords:** Higher education students, Illegal drug-use, Contextual-behaviour change intervention, Psychological Flexibility, Digital intervention

## Abstract

**Background:**

Digital harm-reduction interventions typically focus on people with severe drug-use problems, yet these interventions have moderate effectiveness on drug-users with lower levels of risk of harm. The difference in effectiveness may be explained by differences in behavioural patterns between the two groupings. Harnessing behavioural theories to understand what is at the core of drug-use behaviours and mapping the content of new interventions, may improve upon the effectiveness of interventions for lower-risk drug-users. To the best of our knowledge, this is the first study to systematically apply the Behaviour Change Wheel (BCW) approach to understand the components, influencing capabilities, opportunities, and motivations (COM-B) of higher education students to change their drug-use behaviors. It is also the first study which identifies specific patterns of behaviours that are more responsive to harm reduction practices through the use of the Theoretical Domain Framework (TDF).

**Methods:**

We employed an explanatory sequential mix-method design. We first conducted an on-line survey and a Delphi exercise to understand the factors influencing COM-B components of higher education students to change their drug-use. Subsequently, we mapped all evidence onto the COM-B components and the TDF domains to identify clusters of behaviours to target for change, using a pattern-based discourse analysis. Finally, a series of multidisciplinary group meetings identified the intervention functions—the means by which the intervention change targeted behaviours and the Behavioural Change Techniques (BCTs) involved using the behaviour change technique taxonomy (v.1).

**Results:**

Twenty-nine BCTs relevant to harm-reduction practices were identified and mapped across five intervention functions (education, modelling, persuasion, incentivization, and training) and five policy categories (communication/marketing, guidelines, regulation, service provision, and environmental/social planning). These BCTs were distributed across eight identified saturated clusters of behaviours MyUSE intervention attempts to change.

**Conclusions:**

The BCTs, identified, will inform the development of a digitally delivered behaviour change intervention that focuses on increasing mindful decision-making with respect to drug-use and promotes alternatives to drug-use activities. The findings can also inform implementation scientists in applying context-specific harm-reduction practices in higher education. We present examples of how the eight identified clusters of target behaviours are mapped across the COM-B components and the TDF, along with suggestions of implementation practices for harm reduction at student population level.

**Supplementary Information:**

The online version contains supplementary material available at 10.1186/s12954-021-00491-7.

## Contribution to the literature

- Proposes a conceptual map of the influences of drug-use behaviours in higher education settings
- Suggests an implementation paradigm shift on how to address harms from drug-use among higher education students
- Indicates foci for harm reduction implementation practices in higher education settings

## Background

Illicit drug-use is becoming a public health concern among young adults [1]. The prevalence of drug-use reaches its peak among 18–25 years olds [2–4], with cannabis being the most common illicit drug used by more than 30% of higher education students in the US [5], followed by non-prescribed use of prescription medications, including drugs such as amphetamines [4]. Drug use among young adults coincides with the time when many individuals enter higher education and when experiencing neurodevelopmental processes in the prefrontal cortex that affect risk taking behaviours [6]. Drug use occurs during a period of transition for young adults who are gaining independence, with many living away from family for the first time [7]. In this transitional phase, experimentation with or regular use of drugs is seen as a normative behaviour by students, to either achieve some form of personal enhancements [8], develop new social support structures [2], or enhance a new experience [9, 10]. A period of drug use experimentation can sometimes drive individuals to form new drug-use habits, some of which can continue far into adulthood, leading to possible negative consequences in adult life [11–13].

The potential risks and adverse consequences of drug-use to student populations map across several life domains, including lower grade point averages, poor class attendance [14], heavy drinking [15], polydrug-use [16], and other high-risk behaviours, such as driving under the influence, unprotected sex, physical fights [17], or exacerbation of mental health problems [18]. These consequences may be experienced concurrently, potentially reshaping trajectories of wellbeing across the life course [14, 19, 20]. Given the potential harms illicit drugs can cause, preventive and intervention programmes at higher education institutions are needed to respond effectively to drugs, used by students.

Harm reduction interventions to reduce injection-related harms among young population exhibit moderate-to-large effect for individuals with severe drug use problems [21]. For the population with less severe drug use problems, traditional harm-reduction interventions do not show such a strong evidence of effectiveness [21–24], including when these interventions incorporate personalized information feedback provision and corrections of norms; two components considered to be associated with reductions in harms in other areas, such as alcohol use. Even when these intervention are delivered, digitally, at low-cost, with high levels of acceptability among young adults [25, 26], they achieve only modest success in harm reduction [27, 28]. The reasons for this are the lack of a unified theory-driven behaviour change framework that informs the design and development of the behaviour change intervention. Also, these interventions lack theory-based contextual-driven approaches that incorporate skills training as vehicles to implement harm-reduction practices [21, 27, 28]. In order to effectively employ behaviour change practices, we first need to describe and understand both what is at the core of the target behaviour to change [29] and the contextual influences on this behaviour [30, 31]. We must also make use of process-based behavioural change practices [32–34] which can predict and influence behaviours (e.g. ongoing decisions and actions) with scope, precision, and sensitivity to the context where these behaviours occur [35].

For drug-use, there have been calls for research that goes beyond understanding the antecedents and consequences of use (e.g., motivation for use, perceived harms/benefits of drug use) and towards the explicit use of theories to understand what is at the core of drug-use behaviours [36] within a person’s context [35, 37, 38]. The psychological theories in motivation for change and transtheoretical models of change [39] have produced a plethora of perspectives that explain the phenomenon of drug-use. However, a synthesis of this knowledge to systematically map drug-use behaviours in the users’ context, thus, inform current interventions development and implementation practices, has yet to be developed. Given that harm reduction theory inherently acknowledges that some drug use is likely within different populations [40], innovations in harm-reduction approaches at student population level should target to reduce drug use and minimize the harms occurring through the use of drugs [27, 28]. One framework that can provide a systematic way to use behavioural theories in developing such an intervention is the Behaviour Change Wheel (BCW) [29].

At the core of the BCW approach is the COM-B model [29, 41] which suggests that individuals need the capability (C), opportunity (O), and motivation (M) to change behaviours. In addition, there are nine intervention functions (e.g. modelling) via which an intervention exerts its effect and seven policy categories (e.g. regulation) that support the implementation of the intervention [29]. Also, the COM-B approach includes the behaviour change taxonomy (BCCTv1) [42] which allows the identification of the “active ingredients” of the intervention through a list of 93 possible theory-driven behaviour change techniques (BCTs). Coupled with the COM-B model is the Theoretical Domain Framework (TDF) [31]. This framework consists of 14 theoretical determinants (e.g. knowledge, skills, beliefs about consequences, etc.) which can enhance understanding of the cognitive, emotional, social, and environmental influences on target behaviours [31]. In turn, this knowledge can be translated into agile and effective behaviour change components [36, 43] that can reduce the harm of drug-use at a population-level [44]. In drug-use, the BCW approach and the TDF framework have guided the development and implementation of interventions that target drug-use related harms [45]. But have not yet been applied in the context of higher education populations.

To address this gap, we established the MyUSE(My Understanding of Drug Use Experiences) [46] project that aims to develop a theoretically based, digitally delivered behaviour change intervention to reduce harms from drug use in higher education students. MyUSE project adopted the BCW [29] approach that allows intervention researchers to understand the target behaviours via a behavioural analysis of the problem and then design an intervention on the basis of this analysis.

In this article, for the first time in the relevant literature, we use the BCW approach and the TDF framework, as guides to identify the BCTs that informed the MyUSE content. The specific aims of this study were to synthesize the evidence from primary (survey) and secondary (three systematic reviews) data sources, gathered as part of the overall project, called MiUSE (My Understanding of Drug Use Experiences) [46] in order to (a) identify clusters of drug-use target behaviours among higher education students; (b) select specific intervention functions through which the intervention will exert its effect, and (c) choose specific BCTs to be operationalized within the MyUSE digital intervention.

## Methods

In January 2020, we established a multidisciplinary advisory team with experts in Behavioural Science (VSV, SD2, CL), Information Systems (CH), Public Health (SD1, MD), and Student Health Services (BM) to assist the completion of the BCW approach. The advisory team completed four open-sort grouping exercises, a Delphi-type exercise with two rounds, and a discourse pattern-based analysis, over eight-months to complete the BCW analysis of drug use behaviours. Prior to beginning the analyses, all members attended a training session on the BCW approach and consented to participate in a series of consensus-type meetings. Ethical approval was granted from the Social Research Ethics Committee and University College Cork (UCC) (SREC reference number no: 2018-072A). Written informed consent was obtained from all participants in this study. TIDieR checklist was used for developing this manuscript.

### Phase I—Identifying the problem in behavioural terms

As presented in the BCW approach [41], phase I consisted of four steps. In steps one to three, we analysed drug-use behaviours among higher education students in behavioural terms. In step four, we sought to identify what specific aspects need to change for targeted behaviours to occur.

#### Step 1—Define the problem in behavioural terms

The research group had previously conducted three systematic reviews [27, 28, 48] to identify, gather, and understand all relevant research in relation to the MyUSE project. The first systematic review examined the effectiveness of digital behavioural change interventions for drug use harm reduction in student populations, showing only modest success of these interventions in reducing the harm of drug use [27]. The second review was conducted to examine whether previous similar interventions had employed user-centered design (UCD) practices to inform the intervention development. Findings showed only limited consideration had been given to the end user experience (UX) in designing interventions through UCD practices; limiting their potential effectiveness and sustainability [28]. Finally, the third review examined motivational factors for students’ decision to lower or cease drug use, showing that the identification of the adverse consequences of drug use is not sufficient to prompt behavioural changes among this cohort [48].

Specific findings from these three systematic reviews were used to define the problem in behavioural term and later (step 2) to help us identify items as relevant to targeted behaviours to change. From the first systematic review, an important finding that guided our decision making was that we identified eight studies; of those four focused on cannabis use changes, three on multiple changes in health behaviours and one on changes in different drug use; making it difficult to isolate the mechanism of change. In the second review, we identified personalization and feedback provision as components that can drive behavioural changes. Finally, the third review indicated social factors as strong predictors of drug-use related behaviour, including students’ concerns on how their peers would view them and the feeling of shame surrounding drug use. Both were regarded as important components in defining the targeted behaviour to change.

The advisory group participated in the first open sort grouping exercise, setting out the findings from the three systematic reviews in a template (whiteboard in a class). They worked in pairs and asked to complete a worksheet, focusing on two questions: (a) what is the target group of individuals involved in the behaviour? and (b) where does the behaviour occur? A consensus meeting followed and agreement on how to define the problem in behavioural term was reached.

#### Step 2—Select the target behaviour

The first author (VSV) worked through the findings from the three systematic reviews [27, 28, 48] and the relevant literature to create a long list of items deemed relevant to the targeted behaviour(s). The group then participated in a Delphi-type exercise with two rounds. In each round, the members rated each behaviour using the APEASE criteria (Affordability, Practicability, Effectiveness, Acceptability, Side effects, Equity; see Table 1 for definitions of the APEASE criteria) [47]. The members of the expert advisory group rated the long-list of the targeted behaviours (n = 57), and these ratings were used to collate a shorter-list for the second round of ratings. Inter-rater agreements were calculated, using a 70% threshold as an agreement point for the first round (long-list rating; i.e. participants fall within two agreement categories on a Likert-type scale from 0 = not at all impactful to 5 = extremely impactful [49]), and a threshold of 3.25 median score in the second round (short-list) to resolve differences (convergence biases of opinion) [47]. The final selection of the target behaviour was agreed upon by the expert group members in a consensus meeting.

**Table 1:** Behavioural specification of the target behaviours

Target behaviour	Who	What	When	Where
Increase students’ behavioural awareness regarding their decision to take drugs Reflect on alternatives to drug-use activities which fulfil personal enhancements	Higher education students (aged 18–25 yrs) Full-time/ part time students, Erasmus/visiting students (bachelor to graduate studies)	1. Pause automatic/habitual decision making in relation to drug-use 2. Increase behavioural awareness in relation to drug-use decision making 3. Committing in specified goals which can fulfil personal enhancements	1. In night outs/ social media/ festive periods 2. During induction weeks 3. Before, during & after the exam’s periods 4. Randomly repeated during the semester	Social contexts involving a decision to take drugs (e.g. at campus, in social media, streets around the university, etc.)
Selected items derided from the Delphi-type exercise using the APEASE criteria				
[A1] Users to compare their own use with the norm (descriptive norm correction; e.g. % of students with higher use than their own)				
[A2] Users to reflect on their close friends’ perception about their own use or abstinence (injunctive norm correction)				
[A5] Users to find out personally relevant primary motives for use, listing the personal enhancements drug use fulfil to them (underlying needs that drive the decision to take drugs)				
[A10] Users to reflect on the possible negative consequences of using drugs on academic, athletic and social performance				

Note 1: Given that the four selected items (see Additional file 1: Table 2) are all clustered within the behavioural domain A, specifications are applied for both targeting behaviours

Note 2: The advisory group consulted the APEASE criteria to guide their decisions making. The APEASE criteria specify as to: (a) the impact of the selected behaviour on the intervention’s desired outcomes; (b) the likelihood of behavioural change (c) the spillover effect of behaviour on related behaviours; (d) the accessibility of the targeted behaviour; and (e) the efficacy of the behaviour in bringing about the desired outcomes

#### Step 3—Specify the target behaviour

Following the synthesis of the reviews [7–9], in Step 3 the expert advisory group participated in a third open sort grouping exercise to specify: the target behaviours, the population, and the context (when and where the behaviours will be performed).

#### Step 4—Identify what needs to change

In step 4, we triangulated data to better understand what needs to happen for the target behaviour change to occur [41]. We utilized an explanatory sequential mixed-method design, as defined by Creswell [50]. In this process, we analysed and mapped the data from a quantitative on-line survey of student drug use, onto the COM-B model and TDF framework. The findings from this quantitative analysis informed the qualitative synthesis that followed (the pattern-based discourse analysis [51, 52]). We used the comprehensive set of theoretical construct domains (TDF) to select the domains most relevant to the targeting behaviours and then mapped those domains into the COM-B summative components.

In the first phase, we developed a survey to measure individual and contextual factors relevant to drug-use behaviours (see the Additional file 1; MyUSE drug use survey overview). A Public and Patient Involvement (PPI) group consisting of higher education students reviewed the survey for cultural context appropriateness. The clarity, acceptability, and relevance to students’ context were piloted by a small group of students (*n* = 6) that made several modifications to items and phrasing in the survey. The final survey comprised of six sections (demographics, student life, patterns of drug-use, decision-making process, motivations for using, and behaviour change). The survey was then distributed electronically to a randomly selected representative sample of UCC students (*n* = 3770) via emails. The survey achieved a 30% response rate (*n* = 1138 responses) and a 66% completion rate. Following data clearance, descriptive analysis and reporting (SPSS, V22), a mapping exercise was used, to synthesize qualitatively, aspects of the targeted behaviour to change.

In the second phase, we used the on-line survey data through a large mapping exercise, following a pattern-based discourse analysis [51, 52]. We followed the Strauss and Corbin [53] procedure through a systematic analysis of the on-line survey data, generating categories of intervening (situational factors [54]) that were then translated into narrative statements [53]. Next, pattern-based discourse analysis was conducted in four steps. All derived statements were “grounded” and shaped by university students with previous drug use experiences (theoretical sampling; [54]).

In the first step (open coding), two members of the expert advisory group (coders;  VSV, SD2) identified items from the on-line survey and mapped them in one of the three COM-B components, based on definitions of the components, provided by Michie et al. [41]. The items were analysed quantitatively (descriptive statistics), and then, qualitative descriptors were added to explain an aspect of the targeted behaviours through the COM-B lens [55]. During this process, the coders analysed the selected on-line survey items and extracted patterns (descriptors). In the second step (axial coding), the sequential mixing of data [56] occurred. Here, the coders considered the three COM-B components as the phenomena to focus, and the TDF as the main categories, assembled around the core phenomena [53]. The coders coded the data deductively, assembling the qualitative descriptors, generated previously, in the COM-B components, and then systematically relating these descriptors into the TDF categories, based on the definitions of the domains provided by Atkins et al. [31]. In the third step (selective), the coders selected codes that had been previously qualitatively described (descriptors), to develop narrative statements describing what needs to change for the targeting behaviours MyUSE intervention attempts to change [53]. In the final step, according to Strauss and Corbin [53], the advisory group participated in a meeting, to develop a template with narrative statements (clusters of behaviours to change) that elucidate the potential influences the COM-B and the TDF domains may have in the  MyUSE intervention.

Credibility of the analysis was ensured through multiple mechanisms. The first author, a qualified Clinical Psychologist with experience working with young adults and drug addiction, immersed himself in the whole research process, engaging in an ongoing familiarization with the data and discussions with co-researchers and the Student Advisory Group (SAG) [57]. The second co-author, a behavioural scientist with experience in developmental psychology, examined the patterns for transparency issues [58] and reached agreement on the mapped items across the four steps of the analysis. Thirdly, coders included a detailed log with the coding data [51]. Finally, there were several opportunities for the multidisciplinary advisory team, involved in the BCW approach, to debate and reach consensus in relation to the iterative emergence of the findings.

For each one of the analytic steps of the discourse analysis (open, axial, selective, and paradigm coding), the coders kept thorough memos, writing down ideas and coders’ personal impressions about the emerging narratives, acknowledging the role of reflexivity [57]. In relation to reflexivity, two members of the team (VSV and SD2) led the data interpretation and synthesis part of this analysis. Finally, in keeping with Fine’s recommendations [59] that researchers move beyond the data, we sought to validate the narratives by conducting another qualitative inquiry engaging non-users and users in a card-sorting exercise. Here, we examined the narratives in terms of their importance by presenting a series of cards and asking participants to engage in an open dialogue about their importance in relation to targeted harm-reduction and prevention practices [60].

This mapping exercise was undertaken to examine: (a) what function each TDF domain and COM-B components serve in the target behaviours and (b) what needs to change for the target behaviour change to occur.

### Phase II: Identifying intervention options

Phase II consisted of two steps to guide our decision on intervention functions and policy categories.

#### Step 5 & 6—Identify intervention function and policy categories

Using the APEASE criteria, three co-authors (VSV, SD1, MD) examined, first individually, and then as a group, the nine intervention functions. The goal was to examine whether the functions would serve each of the identified clusters of target behaviours. Further, they also identified policies that support the intervention functions. In each selection of functions, Fleiss’ kappa statistics [61] were calculated, to quantify the reliability of agreement between the raters.

### Phase III: Identify content and implementation options

#### Step 7—Identify behaviour change techniques

To identify specific BCTs, the first author employed the taxonomy matrix of 93 BCTs (BCTTv1) [42] to generate an extended list of BCTs that could be mapped on the eight identified clusters of techniques relevant to drug-use behaviours. Three members of the advisory group with expertise in behavioural science used the APEASE criteria to exclude non-relevant BCTs with the targeting behaviours to change. A shorter list of BCTs was generated, and a final selection of the targeted BCTs was consensually agreed.

#### Step 8—Identify modes of delivery

The mode was predetermined as digital, so this step was not executed.

## Results

**Fig. 1:**
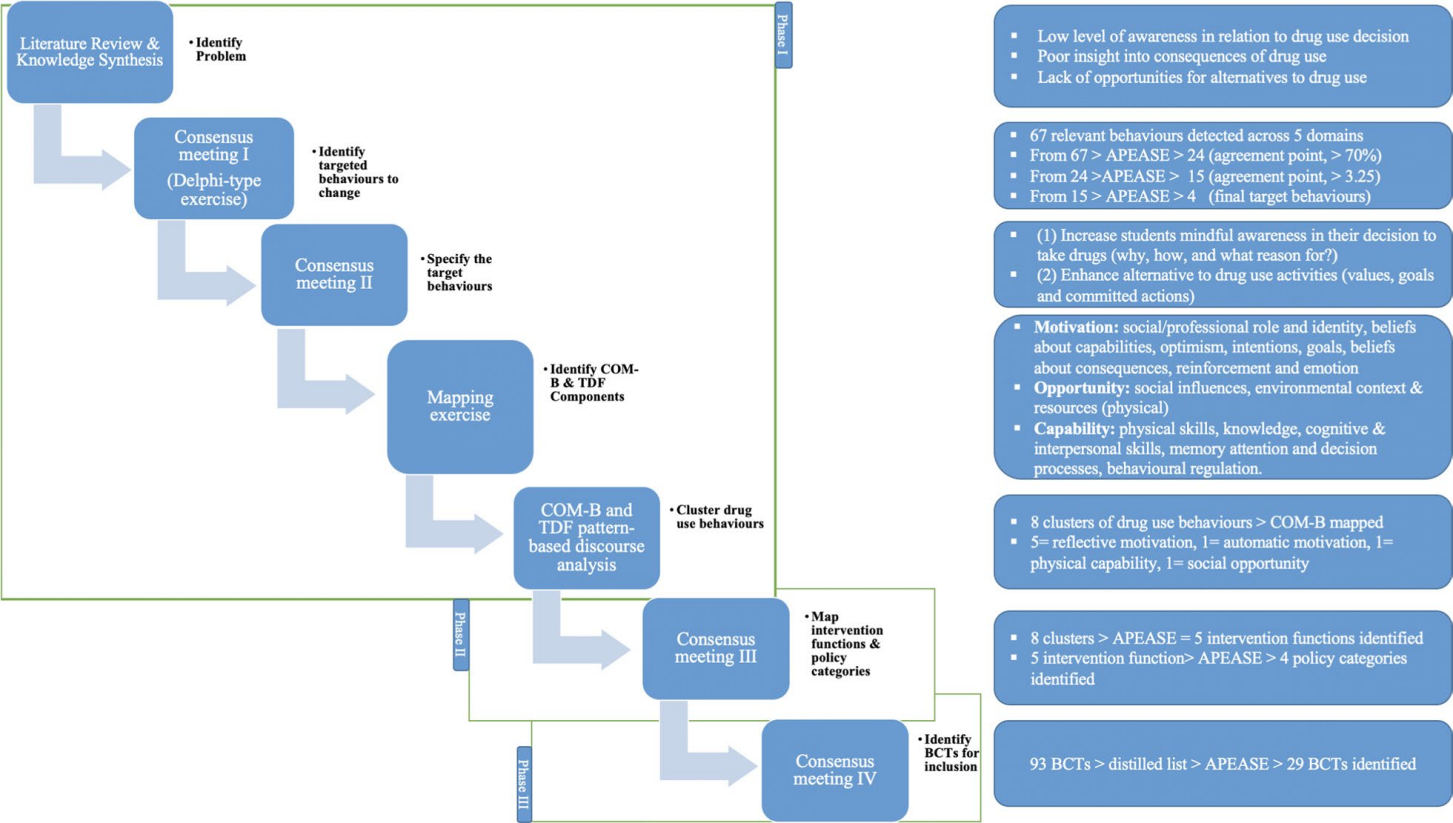
An Illustration of the systematic process of identifying the BCTs

The results reported represent the application of the BCW approach. A summary of the BCW approach employed is illustrated in Fig. 1.

### Step 1—Define the problem in behavioural terms

Following a consensus meeting, the problem was defined as (a) the low level of awareness regarding drug-use decision making, (b) poor insight as to the consequences of drug-use behaviours, and (c) the lack of opportunities to consider alternatives to fulfil personal enhancement. Therefore, the group decided that the intervention should focus on (a) increasing mindful decision-making in relation to illicit drug-use behaviours, and (b) enhancing individuals’ insight for alternatives to drug-use behaviours as a mean to fulfil the students’ personal enhancement. We also decided that this intervention should be most effective for students declaring occasional drug use or no previous drug use. Finally, harm-reduction practices were deemed more useful in social events (e.g. parties, gatherings, etc.) and in places where alcohol is consumed (e.g. pubs, bars, night clubs).

Some of the identified influences on drug-use are related to the physical and social opportunities that may be afforded by the university context. Other factors are related to students’ reflective motivation (e.g. how their peers would view them, feelings of shame) and automatic motivation (e.g. a desired outcome from the use). Finally, capabilities are not identified in the context of students’ drug-use behaviours. The scoping review [48] identified two contextual variables as potentially risky factors: the university context and the transition period from the second level (i.e. high school) to higher education. These factors require harm-reduction strategies at a systemic/policy-level (e.g. new public health responses to illicit drugs and alcohol use) [62]. However, our analyses of behavioural diagnostics indicated the value of individual-level focus.

### Step 2—Select the target behaviour

**Fig. 2:**
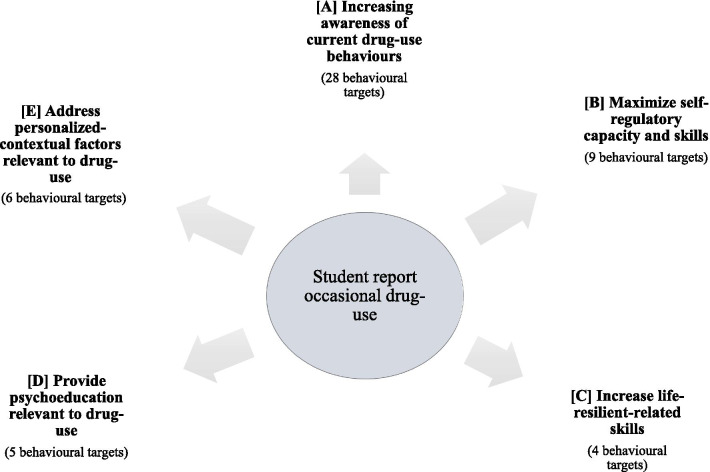
Potential behavioural targets relevant to drug-use

A long list of 67 potential behaviours (items) was derived from the synthesis of the relevant literature. Figure 2 illustrates five potential targets relevant to drug-use behaviours that can increase students’ awareness in relation to their decision to take drugs and can increase understanding of alternatives as means to fulfil the students’ personally relevant enhancements (the entire list is shown in Additional file 1: Table A1). In refining the long list of potential targets, one can see that current university service provisions address some of the targeting behaviours. For example, psychoeducation about the consequences of illicit drug-use (domain C) is one service universities often provide as part of their health care policies [62]. The advisory group participated in a Delphi-type exercise with two ranks.

In round one from the 67 items identified, only 24 items reached the agreement point of 70% (see Additional file 1: Table A1). Seventeen items were from the targeted behaviour A (increase awareness of current illicit drug-use behaviours), four from B (maximize self-regulatory capacity and skill), one from C (resilient-related skills), and two from E (address personalized-contextual factors). None of the items from domain D (provide psychoeducation relevant to drug-use) reached the agreement point, and this behavioural target was excluded from round two.

In round two, from the 24 items selected, 15 items reached the agreement point of the median score, using the APEASE criteria (values > 3.25), as potential drug-use behaviours to target (10 from A; 4 for B; and one from E; see Additional file 1: Table A2). Finally, of these 15 items, only 4 reached the agreement of the mean score of the APEASE criteria (ranked > 70%) and these were selected as potential behaviours to target. All four items (targets relevant to drug-use behaviours) were from the behavioural target A (see Table 1 for the four identified behaviours). These items indicate that the targeted behaviours should increase awareness in relation to contextual factors (e.g. peers) that influence drug-use decision making and enhance insight as to the internal motivations of the students to use drugs (e.g. expectations).

### Step 3—Specify the target behaviour

Table 1 presents the specifications of the targeted behaviours and the four selected items derived from the Delphi-type exercise.

### Step 4—Identify what needs to change

We present an overview of the findings from the on-line survey. We then present the findings from mapping the identified patterns of drug-use behaviours onto the COM-B components. We finally present the findings from the discourse-based analysis that show what needs to change for the targeted behaviours to occur, using the TDF domains. Table 2 below shows the patterns of drug-use related behaviours mapped on the COM-B components and the TDF domains. Table 3 summarizes the findings of the whole analyses arising from the step 4.

**Table 2:**
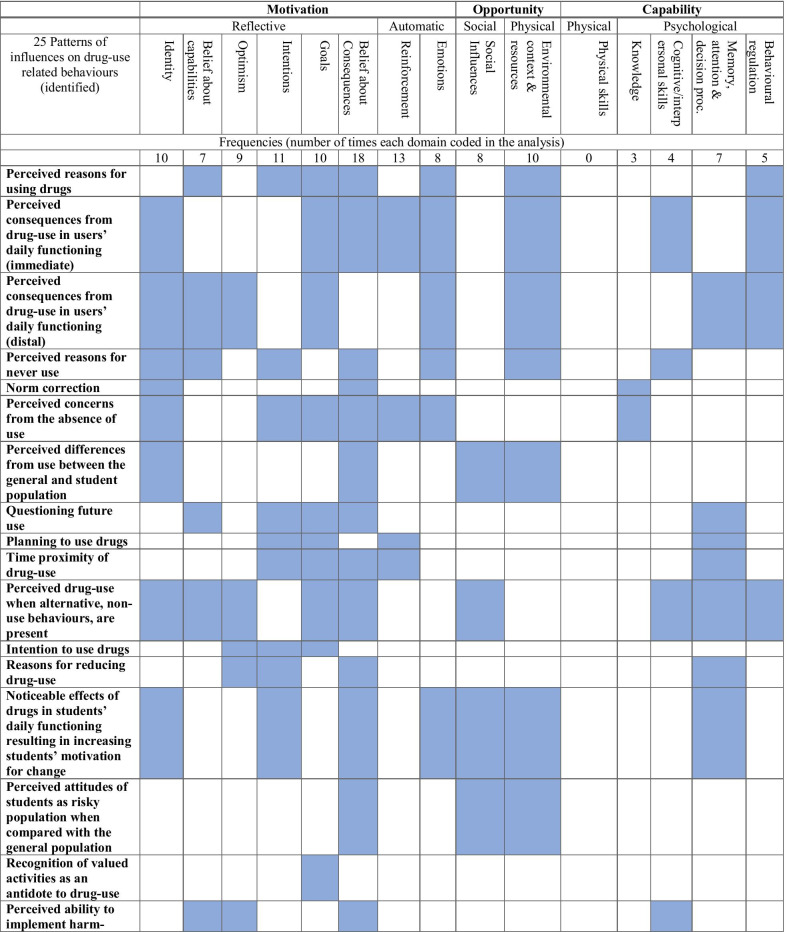

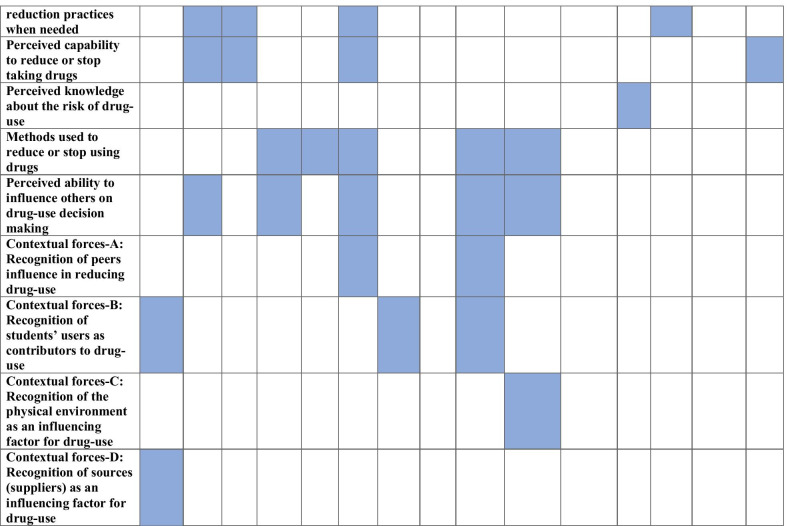
Mapping patterns of drug-use related behaviours within COM-B components and the TDF domains

Note 1: we identified the following TDF domains, expanded on the COM-B components; *motivation* [86]: social/professional role and identity (10), beliefs about capabilities (7), optimism (9), intentions (11), goals (10), beliefs about consequences (18), reinforcement (13) and emotion (8); *opportunity* [18]: social influences (8), environment (10); *capability* [19]: physical skills (0), knowledge (3), cognitive and interpersonal skills (4), memory attention and decision processes (4), behavioural regulation (5)

Note 2: Id.: Social/Professional Role and Identity, Bel cap.: beliefs about capabilities, Opt.: optimism, Int.: Intentions, Bel cons.: Beliefs about consequences, Reinf.: reinforcements, Em.: Emotions, Env.: Environmental context & resources, know.: knowledge, cog.: cognitive and interpersonal skills, mem.: memory, attention and decision processes, Beh. Reg.: behavioural regulation. The shaded squares highlight evidence or consensus that these identifiers map on a specific TDF domain

**Table 3:** Intervention components targeting the eight identified patterns of drug-use related behaviours

Pattern of drug-use related target behaviours	COM-B mapped elements targeted	Intervention Function served	Coded identified (from BCTTv1)	BCTs selected	Translation of BCTs within MyUSE intervention
Cluster 1: Increase awareness about the real vs. anticipated effects drug-use can have on students’ personally desired behaviours	Reflective motivation	Education	5.1	Information about health consequences	Provide a personalized animated feedback explaining the possible health-related risks and harms per drug-use type and frequency
			5.3	Information about social and environmental consequences	Present a gamified quiz showing the possible consequences of drugs in students’ academic and emotional area of living (e.g. legal problems, etc.)
			5.6	Information about emotional consequences	
			6.3	Information about others’ approval	Provide a subjunctive norm correction, in a gamified quiz way
			13.4	Valued self-identity	Include a story-telling exercise requesting from students to produce self-statements of their life they want while in college
Cluster 2: Promote identification of personally relevant activities (which are they?) which lead to positive expected outcomes in students’ desired behaviours (fun & enjoyment)		Education	1.3	Goal setting (outcome)	Present an interactive value’s identification and committed action exercise
			7.2	Cue signalling reward	Present a reflective- non-judgmental rhetorical question, prompting students to consider how their values match with drug-use behaviours
			13.2	Framing/reframing	Suggest the adoption of alternative to drug-use activities as means to enhance fun and enjoyment
		Modelling	5.6	Information about emotional consequences	Present a mindful-based exercise (“how fully present am I?”), prompting students to visualize (mental representation) how they would feel after achieving college-related goals
			15.1	Verbal persuasion about capability	Use motivational cards to show how students can pursue value committed actions
			16.3	Vicarious learning	Present a scenario-based story showing a student pursuing his/her goals while enjoying college’s years
		Training	1.4	Action planning	Prompt students to use their e-calendar to plan implementing SMART defined goals
Cluster 3: Increase perceived competence & optimism that an implementation plan of alternatives to drug-use activities can induce positive expected experiences (fun & enjoyment)		Education	1.3	Goal setting (outcome)	Use an animation to educate how commitment to SMART goals can work as alternative to drug-use behaviours and reinforce the deliberate adoption of several, non-drug-use behaviours, asserting that these behaviours can elicit as fun as drugs can, without putting students into risks or harms
			1.9	Commitment	
			6.3	Information about others’ approval	
			8.2	Behaviour substitutions	
			8.6	Generalization of a target behaviour	
			13.2	Framing/reframing	
			15.1	Verbal persuasion about capability	
Cluster 4: Increase awareness of the university context as a risk factor that increase the chances for drug-use behaviours and how this can restrict students from having positive college’s experiences		Education	2.3	Self-monitoring of behaviour	Explain the role of cues (triggers of use) within an ABC analysis (behavioural analysis), prompting students to identify (self-monitoring) their own antecedent triggers in relation to the context of use
			4.2	Information about antecedents	
		Modelling	7.1	Prompt/cues	Show how self-talk can be used to help students recognize cues that can influence decision making in relation to drug-use
			15.4	Self-talk	
Cluster 5: Cultivate mindful awareness of the perceived reasons for using (why I use?) and increase insight as to whether the use leads to desired outcomes in goal-directed behaviours at a long-run		Education	1.6	Discrepancy between current behaviours and goals	Present a personalized feedback showing students’ level of behavioural awareness and goal attainment (i.e. whether their decision to take drugs is influenced by others). Then, prompt students to monitor whether there is a discrepancy between goals and present behaviours
			2.4	Self-monitoring of the outcomes of behaviours	
			4.1	Instructions on how to perform a behaviour	Introduce via an animation the mindful decision-making skill (learn to pause step-back, notice and decide) and prompt students to think how this skill resonates with their role identity
			8.1	Behavioural practice/rehearsal	
			13.4	Valued self-identity	
			4.2	Information about antecedents	Prompt students to apply the new skills to identify cues
		Modelling	4.1	Instructions on how to perform a behaviour	Present via a story-narrative (a party house) how a student applying mindful decision-making skill (pausing- noticing- deciding) in a situation requiring drug-use decision making.@@Reinforce students’ capacity to apply the new skill in different situations
			6.1	Demonstration of the behaviour (modelling)	
			8.1	Behavioural practice/rehearsal	
			8.6	Generalization of a target behaviour	
			15.1	Verbal persuasion about capability	
		Training	6.1	Demonstration of the behaviour	Present a series of mindful cards demonstrating what mindful decision-making skill does and advice for a gradual implementation of this skill building activity
			8.7	Graded tasks	
Cluster 6: Resolve students’ misleading expectations about the expected outcomes of drug-use in students’ desired behaviours in the long-run	Automatic motivation	Education	5.2	Salience of consequences	Provide a general performance score in a drug-use quiz activity. Wrong-answered responses will provide feedback that will target at correcting students’ expectations about the effect of drugs in the long-run. They will also aim at increasing awareness about the potential regrets, students may experience from the use of drugs
			13.2	Framing/reframing	
		Persuasion	5.5	Anticipated regret	
Cluster 7: Increase procedural knowledge and practice skills on how harm-reduction practices are implemented within the university context	Capability physical	Education	1.8	Behavioural contract	Present a series of harm-reduction practices and invite students to select the ones that best fit with their experiences (personalized plan)
			4.1	Instructions on how to perform a behaviour	Show instructions in detail on how to perform selected harm-reduction practices via a series of illustrative cards
			8.6	Generalization of the target behaviour	Prompts students to generalize the new harm-reduction practices, including awareness of exposure to cues, to multiple different situation
			12.3	Avoidance/ reducing exposure to cues for the behaviour	
		Modelling	1.9	Commitment	Show an animation illustrating a student to perform one harm reduction practice, highlighting his/her commitment
			4.1	Instruction on how to perform a behaviour	
		Training	4.1	Instruction on how to perform a behaviour	
			6.1	Demonstration of the behaviour	
Cluster 8: Promote behavioural awareness and behavioural regulation regarding drug-use decision making under the influence of peers	Social Opportunity	Education	1.6	Discrepancy between current behaviour and goal	Prompt students to reflect on their personalized feedback scores in questionnaires assessing levels of decision making influenced by others
			4.1	Instruction on how to perform a behaviour	Present instructions on how to promote behavioural awareness in relation to decision making
			4.2	Information about antecedents	Prompt students to think peers’ influences as antecedent (cues) for them to use drugs
			5.2	Salience of consequences	
			8.2	Behavioural substitution	Promote a mindful decision-making, highlighting that influences from peers should be aware
			8.7	Graded tasks	Provide suggestions on building the new behavioural regulation skill (behavioural awareness)
			13.2	Framing/reframing	Prompt students to consider their feedback on subjunctive norm correction (how behaviours are approved by others) in relation to their valued identity
			15.2	Mental rehearsal of successful performance	Advise students to imagine themselves taking a mindful decision, despite the presence of peers’ influence
		Modelling	4.1	Instruction on how to perform a behaviour	Illustrate an animation (a narrative story) with a student denying using drugs, while recognizing the potential influence of peers
			6.1	Demonstration of the behaviour	

Note 1: Physical opportunity was not targeted

Note 2: Translation of BCTs into digitally delivered components represents only concepts which are designed into ideations prototypes to be tested with students, rather than actual implementation practices for the MyUSE digital intervention

#### Findings from the on-line survey

Almost a third (32%) of respondents reported using an illicit drug in the last year (*n* = 236; current users) with 44% reporting no previous use of illicit drugs (*n* = 324). Cannabis was the most commonly reported drug (*n* = 230; 31.25%), followed by ecstasy (*n* = 139; 19%), cocaine (*n* = 120;16.30%), ketamine (*n* = 73;10%), mushrooms (*n* = 53; 7.20%), and others (*n* = 121; 16.55%). The age of first use was 19–21 years for most drugs, except for cannabis which was 16–18 years old.

The majority (> 77%) of respondents indicated experiencing negative effects from the use of any drug. They reported motivations to abstain from concerns raised regarding the impact of drugs on their psychological well-being, cognitive function, academic performance, and the lack of further pleasuring effects. The majority of responders (82%) also believed that students are much more likely to use drugs, compared to the general population, mostly due to opportunities for use, provided by the university context (e.g. acceptability, lack of control, and peer influences). Students reported social factors related to use, including peer pressures (54%) and at least one occasion (reported by 81%) where they were around people who were using drugs. The majority of students (72%) reported that they would be positively influenced to abstain if their friends reduced their usage. The primary reason for use was given as “fun and enjoyment” (86%), followed by “coping with daily academic stressors” (7.3%).

Students felt they possessed adequate knowledge of the risks associated with drug-use (89%), mentioning perceived deterioration in finances (9.5%), personal physical safety (42%), academic progress, physical activity (40% in both conditions), athletic performance (35%), and psychological wellbeing (32%) as the main areas that are affected by drug-use behaviours. Notably, students reported experiencing positive changes in several areas of functioning while taking drugs, including increases in confidence (95%), social interaction (92%), relaxation (86%), energy levels (62%), and decreases in irritability (70%), and distress (68%), with these effects reverting when the effects of the drugs wear off. For those declaring previous use, the five main motivations for change were: noticeable psychological impacts, financial burden, physical effects, impairments in executive functions, and concerns about how other people perceive their drug-use. Students also reported willingness to change their use if they were to socialize with other groups (18.7%), had alternatives to drug-use activities (24%) or had better ways to manage unwanted emotions (10.5%). From those declaring current use, 41.3% reported no confidence to use harm-reduction measures as means of protecting themselves from the effects of drugs, with 36.6% reporting “somewhat confidence” and 22.1% “confidence”.

#### Findings from the pattern-based grounded discourse analysis

During the first step of the coding (open coding), we identified, coded, and mapped onto the COM-B a total of 25 patterns of drug-use behaviours derived from the survey (see Table 2). 23 items were coded into motivation (17 reflective and 6 automatic), 5 in capability (3 in physical and 2 in psychological), and 7 in opportunity (5 in social and 2 in physical). Most of the selected items were first analysed quantitatively and then described, qualitatively (see Additional file 1: Table A3; step 1). They all focus on the role of reflective motivation. Data indicate how students’ behaviours are habitually driven by their motivation to take drugs in order to achieve a desired end-goal which is always relevant to college's life (e.g. have fun, etc.). This decision occurs automatically and is partly grounded in the absence of students’ awareness of  other means that lead to similar outcomes in desired behaviours, but with less risk of harms for them (e.g., valued-drive activitied). Though descriptors provide salient categories -indicating students’ increased awareness about the risks and harms of drugs, what prevails is a habitual and automatic decision making on behalf of students, particularly when drugs are involved in their decision. Further, analysis showed that social opportunities increase decision making, favouring drug use. Finally, although the analysis showed sufficient psychological capability (i.e. knowledge, understanding) from students to reduce the use or harms from drugs, poor knowledge about how harm-reduction practices can be applied, limit capabilities towards the targeting behaviour.

In the second step of the analysis (axial coding), as presented in Table 2, we fit the descriptors of the initial coded data into the central phenomena (COM-B components) and the main categories (TDF). We synthesized the coding data into selective qualitative descriptors (see Additional file 1: Table A3; step 2). In the third step (selective coding), we developed narrative statements, based on selective codes, exploring the role of each TDF domain (descriptor) in influencing the three behaviours MyUSE attempts to change (see Additional file 1: Table A3; step 3). In our analysis (see below), we examined what factors support the three targeting behaviours within each TDF domain, and also what competes or inhibits the desired behaviours to change.

#### Reflective motivation

Beliefs about consequences: students have strong expectations of the role of drug-use in enhancing personally relevant areas of interest. The immediate and potent effects of drugs (e.g. increased energy level, social interactions, confidence, reduction in anxiety, irritability, etc.) enhance students’ beliefs as to the long-term effects of drugs in fulfilling areas that matter the most to them (e.g. having fun with their friends). Although students report having concerns about the negative effects of drug use in the long-term (i.e. more than 80% agreed on that), these beliefs are buffered by the strong and immediate positive effects drug can have in students’ relevant areas of interest. Further, the immediate effects from drug use are directly and indirectly reinforced by contextual variables (peers, perceived expectation for use in higher education, fun, etc.), disinhibiting the potential effect of any negative beliefs students have in the long-run. This leads students to maintain misleading expectations as for to the effects of drug use in the long-term. Therefore, increasing students’ awareness of the perceived long-term consequences versus the perceived short-term benefits may lead to an increase in students’ harm reduction practices and possibly reduces levels of drug-use.

Intention: the current users’ intention to abstain from drug-use, in contrast to the non-users, was found to be low. Students report confidence to use harm-reduction practices, selecting the ones that they consider as the most effective ones (e.g. avoid certain environments or people who frequently use). However, the use of harm-reduction practices is buffered under the presence of contextual influences that prevail. Low behavioural awareness, lack of planned alternatives, and long-term habitually established patterns of drug-use behaviours are theorized to lower students’ motivation to engage in preventative or protective health behaviours. Harm reduction interventions should help students identitfy personally relevant valued-based activities and highlight the role of drugs in disrupting the completion of valued-based activities.

Social/professional role and identity: although students present with sufficient awareness of the negative effects of drug-use in their social identity (e.g. academic disruptions, risks in physical safety, reductions in popularity levels, etc.), contextual forces (e.g. acceptability of drug-use, peer pressure, fear of not fitting in, etc.) undermine the effect of this awareness on students’ motivation to change (e.g. protect themselves, abstain or reduce the use). Increasing awareness of the negative effects of drugs on students’ identities (e.g. valued self-identity) can support harm-reduction interventions in higher education.

Goals: The goal of students who take drug is to fulfil some personal desires (e.g. such as induced fun and excitement while in college). This goal can become habitual, forcing students to either plan specific actions to get drugs or prioritize activities around the drug-use. Findings showed that 50% of students plan to use drugs in time proximity (hours shortly before using), and 38% have a conscious plan (goal) several days in advance. Students report a willingness to abstain or reduce their drug-use if alternative activities will help them to achieve certain value-based outcomes (e.g. academic progress, secure physical safety, etc.). Therefore, altering the means via which students reach desired effects in personally relevant areas of interest (e.g. fun) can support harm-reduction interventions.

#### Automatic motivation

Reinforcement: Students’ drug-use (a response) is contingency related with some positive effects (a stimulus) in certain college’s areas of interest (e.g. athletic performance, concentration enhancement, academic achievements). Changing the contingency from having  a specific stimulus (desire to achieve positive effects in certain areas of college’s life; e.g. have fun) and a response (take drugs) to acquire a new stimulus (drugs = risky) and a respond (protect myself through harm reduction practices or cease/reduce drug use), could support harm reduction interventions.

#### Opportunity

Environmental context and resources (Physical): Each contextual factor (e.g. perceived normalization/acceptability of drug-use within university settings, peer influence, venue, etc.) has a linear effect on students’ decision making; as the contextual factors increase, so does the degree of influence for students’ decision to take drugs. A harm reduction intervention should enhance students’ awareness about the “synergetic” effects of environmental antecedents (personal and interpersonal cues) and their role in increasing social opportunities for drug-use.

In the final step (paradigm coding), we organized the information originating from the previous coding step, into eight narrative statements (clusters of drug-use behaviours), coded around the central phenomena of the COM-B components (see Additional file 1: Table A3; step 4). As presented in Table 3, five clusters target reflective motivation, one automatic motivation, one physical capability, and one social opportunity.

### Step 5—Identify intervention functions

Five intervention functions were identified from the eight clusters of target behaviours, using the APEASE criteria. The overall reliability of agreement between the raters was moderate *k* = 0.47 (0.95% CI 33 to 0.60), *p* < 0.001. As seen in Table 4, we selected education, modelling, and persuasion as the predominant intervention functions. To address the possible low engagement with the new behavioural repertoires (skills), we included incentivisation, considering that the expectation of rewards in personally relevant behaviours may have reinforcing effects on the target behaviours. Finally, in response to students’ lack of knowledge about implementing harm reduction practices, training was recognized as an important intervention function, mostly because it promotes procedural knowledge and practical skills (e.g. how to implement harm reduction practices).

**Table 4:**
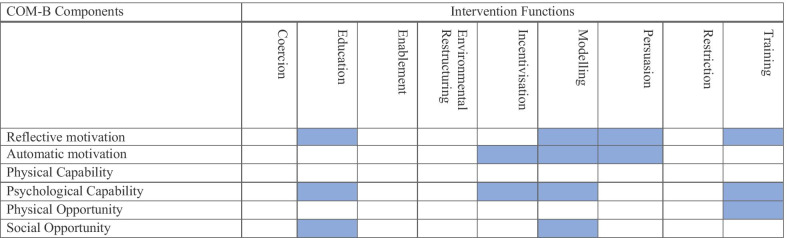
Links between the COM-B components and intervention functions

Note: The shaded squares highlight evidence or consensus agreement among the members of the advisory group and show that the identified clusters of target behaviours (8) can be targeted with a particular intervention function or a combination of them

### Step 6—Identify policy categories

We also decided that five policy categories could serve the five selected intervention functions: (a) communication/marketing, (b) guidelines, (c) regulation, (d) service provision, and (e) environmental/social planning. The first three policies were shared across at least four of the five intervention functions. Environmental/social planning was considered a supporting policy for the incentivisation as an intervention function. Both communication/marketing and service provision policies were selected to support post-design promotional and delivery activities of the MyUSE digital intervention, rather than to update its content.

### Step 7—Identify behaviour change techniques

**Fig. 3:**
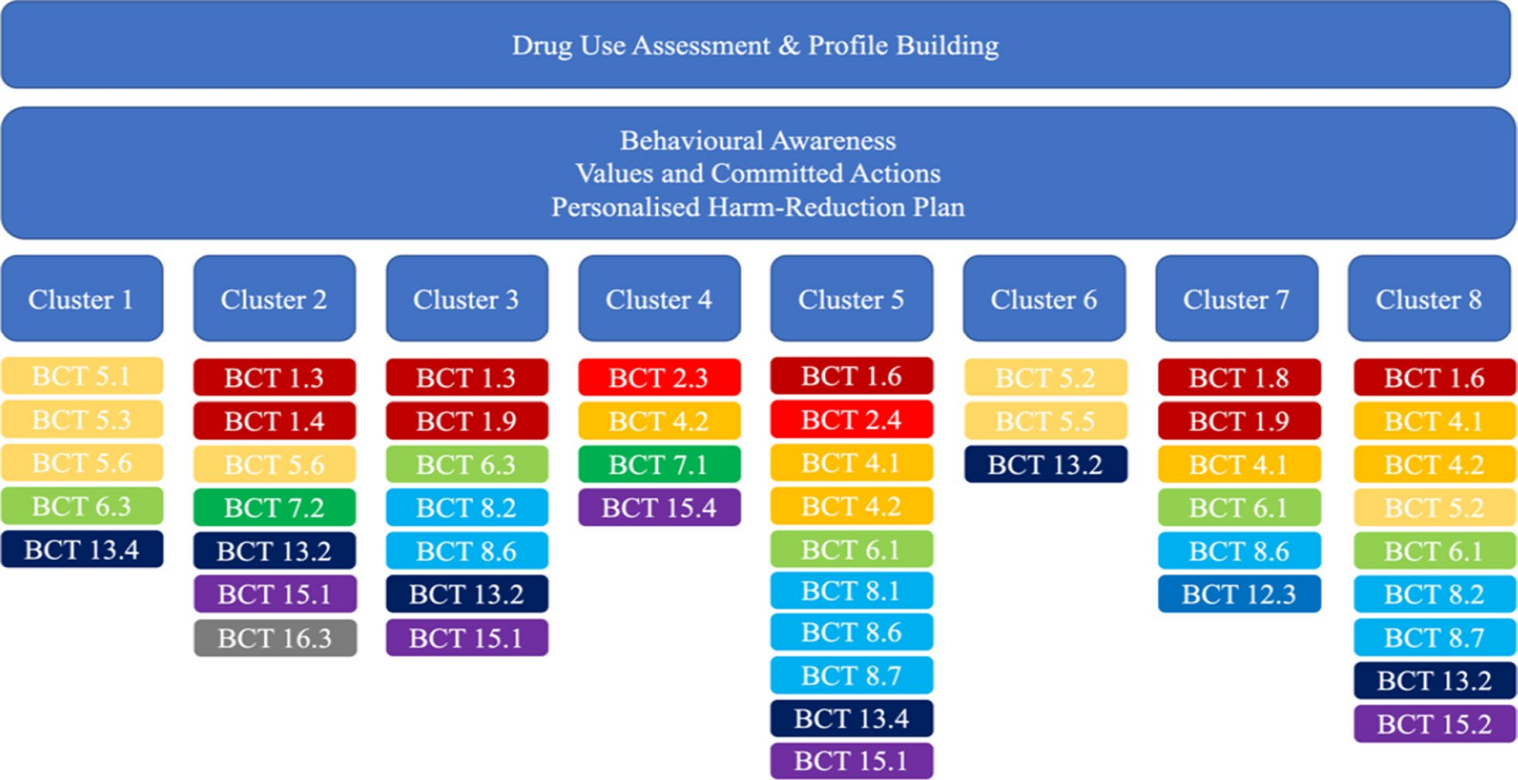
Combination of the selected BCTs matched with the eight clusters of target behaviours. Note1: Clusters: (1) Increase awareness of the effects of drug-use on personally desired behaviours; (2) Promote identification of activities which can have a positive effect on students targeted behaviours; (3) Increase optimism that drug-use alternatives can induce long-term positive experiences; (4) Increase awareness of the university as a risky context for drug-use; (5) Cultivate awareness of the reasons for use and how these lead to students goal-directed outcomes; (6) Resolve expectations about the effects of drugs on students desired behaviours in the long run; (7) Increase procedural knowledge on how harm reduction practices are implemented at a university level; (8) Promote behavioural awareness of the decision to take drugs under the influence of peers. Note 2: Actual names with definitions of the labels (BCCTv1) can be found at Michie S, Atkins L, West R. The behaviour change wheel: a guide to designing interventions. 2014. Great Britain: Silverback Publishing. 2015. Note 2: Boxes with the same colour represent different groups of BCTs as they were organized in 16 groupings during the first version of the BCT Taxonomy v.1

We created a long list of potential BCTs (see Additional file 1: Table A4). Using the APEASE criteria, we identified 29 BCTs matched with the eight clusters of behaviour to change and COM-B components (see Table 3). In Fig. 3, we illustrate the combination of the selected BCTs (BCCTv1) matched with the eight clusters of target behaviours.

## The MyUSE digital intervention

The identified BCTs have led us to develop a multi-component personalized web-based digital intervention. The intervention consists of 11 modules, distributed in three phases: the Allocation phase I (module 1), the Profile building phase II (modules 2 to 4), and the Skill-building phase III (modules 5 to 11). During phase I, the intervention assesses individuals’ drug use history and drug type and allocates users onto one of the following three strands: non-users, low/ moderate users, or severe users. In phase II, individuals build their profile with the risk of harms from drug use and identify areas of skills lacking in relation to harm-reduction practices (see Table 3). During phase III, participants receive a series of personalized skill-building modules, attempting to address their needs, either for harm reduction practices (in case of low/moderate or severe users) or prevention skills (in case of non-using students). For example, individuals may enhance skills attempting to increase mindful decision making in relation to drug use, help them identify their personalized triggers, behaviours, and consequences of their drug use, and motivate them towards developing their own harm-reduction plan (either drug or non-drug related). A new clinical algorithm that is embedded in the system’s interface decision tree logic (see [84]) harnesses individuals’ anonymous personal data to present personalized suggestions, and provide modularized intervention’s components based on their needs.

## Discussion

To the best of our knowledge, this is the first study to systematically apply the BCW and the TDF frameworks to understand the dynamic and complex determinants supporting harm reduction practices in the context of higher education students’ illicit drug-use. The findings have guided the design of the MyUSE intervention through the identification of the contextual, cognitive, and emotional determinants that support students’ decision making to use drugs. The findings also generated a novel comprehensive conceptual map of the influences on drug-use behaviours in higher education students. This conceptual map indicates foci for harm reduction implementation practices and new paradigms in addressing drug-use among higher education students.

Increasing reflective and automatic motivation, physical capability, and social opportunity are important determinants to consider in supporting harm-reduction practices for higher education students. Harm reduction practices that respond to these determinants can be translated into selected BCTs which based on the study’s theoretically grounded hypothesis, may work synergistically to increase students’ mindful decision-making to drug-use, and enhance their motivation that lowers the risk of harm (either drug or non-drug related). The analysis showed that reflective motivation prevails, indicating that any behavioural change intervention should focus on increasing students’ reflective motivation. Yet, several other determinants should be also considered. Correcting students’ expectancies about the benefits of drug-use in the long-run, increasing insight of finding alternatives to drug-use activities as means for fulfilling students’ desires (mostly to have fun and enjoy activities), and enhancing awareness of their personal (e.g. personality, sensation-seeking, emotional dysregulation) and contextual (e.g. peer influences, norm perceptions, etc.) factors that influence students’ drug-use decision are all novel implementation practices that can tackle the harm drugs can cause in higher education students populations.

Findings from the discourse pattern-based analysis identified eight patterns of drug-use behaviours. The eight patterns make use of education, modelling, persuasion, incentivisation, and training as the predominant intervention functions. These functions are supported by communication/marketing, guidelines, regulation, service provision, and environmental/social planning policy categories. Any activity that focuses on cultivating one of the eight clusters of behaviours relevant to drug use should be implemented, using a combination of the 29 identified theory-driven BCTs which fulfil the criteria for interventions being implementable in an affordable, practical, and acceptable way [63]. Notably, BCTs can be used in different modes of delivery, populations, contexts, and relevant types of behaviours [64], and as expected from the TDF framework, can increase clarity as to the mechanisms of action through which behaviour changes occur.

Drawing on the findings from the BCW analysis, this study provides a clear theoretical map for researchers and implementation scientists, highlighting novel context-specific components that can be translated into effective modularized, personalized harm-reduction practices. To achieve this objective, any effort to mitigate the harms drugs can cause in students’ lives, require a multicomponent intervention that takes account of the specific developmental context of higher education and the life stage of students [12, 65]. This study illustrates how this can be achieved through the use of the eight clusters of target behaviours. In combination, these behavioural changes may enhance opportunities for creating positive life trajectories via teaching mindful decision-making and value-based actions. They also focus on increasing motivations for change and enhancing sensitivity to contextual influences and opportunities, including drug availability, environmental triggers, and the most salient features of the educational context [21, 46–67]. To date, previous interventional efforts have received criticisms as being too narrow (e.g. misperceived norms, lack of knowledge about harm-reduction practices, low motivation for change, etc.) or adopting a one-size-fits-all approach (e.g. individuals presenting with different levels of use, non-using students, etc.) [27]. Though these interventions are promising [68], they address context variation and personalization for students, poorly [66, 69]. What is missing are innovations in delivering tailored-made harm-reduction supports to students’ in higher education.

There is a need for use of contextually driven approaches that can deliver greater behavioural regulation by harnessing social, psychological, and situational forces [38, 70]. Of equal importance is the need for multidisciplinary collaboration in developing and delivering such preventive and intervention programmes. While COM-B and TDF frameworks provide a more granular understanding of psychological capability and reflective motivational processes [42], this knowledge can also guide researchers and implementations scientists to other relevant theories and approaches [31]. The findings from the BCW approach identified the key role of students’ mindful decision-making in relation to drug-use and the promotion of valued-based activities; two components originating from the positive psychology strand of the third-wave cognitive behavioural interventions [72, 73].

Contemporary behavioural accounts of psychological health indicate Psychological Flexibility (PF) [35] as a potentially effective construct to support students’ mindful decision-making. PF encourages the disinhibition of immediate habitual gratification (e.g. taking drugs for having fun) over individuals’ long-term goals [38, 72]. It does this by teaching individuals behavioural awareness or the ability to be present and take decisions, considering all the possible influencing factors [74]. Several combinations of BCTs, as presented in clusters #4 and #5, teach individuals how to practice mindful decision making. For example, for those declaring previous use, the skill attempts to teach awareness of the triggering influences of behaviours (e.g. contextual and interpersonal) and the consequences (effects) of them prior to decision making. For those with no-previous use, the skill attempts to cultivate awareness of the potential factors that can influence the decision to abstain. The PF model approach also reinforces the recognition of personally desired life directions in domains that are congruent with students’ values, such as academic achievement and attenuation of personal career goals [38]. Given that targeted behaviour change can be effective, if they adopt specific approaches [42], PF purports to enact behaviours (e.g. coping strategies) that maintain positive trajectories in youths’ lives [72].

Research shows that PF can achieve this goals at higher education [32, 75, 76] and in drug-related behaviours and disorders [77]. This can be achieved by capitalizing on the clinical application of the PF model, coined Acceptance and Commitment Therapy (ACT; [35]) that indicates six therapeutic facets, three of them align to the MyUSE goals: to increase mindfulness, promote the identification of values, and cultivate committed actions [32, 78, 79]. Employing specific evidence-based therapeutic facets, such as the ones the ACT approach proposes [78–80], can increase sensitivity in treatment outcomes and knowledge of the mechanisms via which behavioural changes occur [35]. Finally, conceptualizing harm-reduction practices from a PF perspective can enhance the idea of personal responsibility from a positive psychology perspective [72], as opposed to existing medico-legal perspectives that highlight users’ passivity and risk-taking behaviours [81, 82].

On the ground of these findings, we developed a digital intervention that will “start where the users are at” [83], motivating them to either reduce the harms, lower the use or prevent non-users from potential harms by reinforcing targeted skills. MyUSE approach of harm reduction provides a pragmatic yet compassionate set of skills developed to reduce the harmful consequences and the use itself [40]. This necessitates the development of a multi-component intervention that will equip individuals with tailored skills in identifying, recognizing, and preventing harms before they occur. From a developmental perspective, we believe that these skills will not only help young adults to cope with the effects of drug use, but also, shape behaviours towards value-based and committed actions [72, 73] Therefore, this intervention should be considered as both a primary prevention intervention for those with no previous experience and a secondary intervention for those with previous or current drug use.

Findings should be interpreted in the light of some limitations. Despite the systematic approach of the BCW approach, the triangulation of the present study data analyses occurred without specifying the primary drug-use. Though most higher education students reporting using cannabis, a sensitivity analysis per drug-use type may have revealed influences on behaviours relevant to specific types of drug-use (e.g. different clusters of behaviours from students using stimulants). This analysis could highlight aspects of drug use types and behavioural responding that are perceived as both having positive and negative effects to individuals. Likewise, the findings from the behavioural analysis focus mainly on understanding the drug-use of experimental/occasional-use students which is the most frequent group of using students [2], with limited references to those students who do not use drugs or those who describe heavy drug use. This limits interpretation of the present study findings to non-using or severe using populations, warranting further research. Further, the interrater reliability for the selection of the intervention’s functions was moderate, limiting interpretation of the selected intervention function. Finally, given that no well-validated scales were employed to capture the key indicators of the COM-B components in the survey, the findings may include measurement biases and so replication may be worthwhile.

Future research should assess which identified clusters of behaviours may improve efficacious outcomes if a change is needed. This should be conducted prior to pilot feasibility or pragmatic implementation trials to avoid premature development of a full service which may later need significant modifications. In addition, future research should examine how selected clusters of behaviours could be effective when applied in different contexts (e.g. digital, public health policy practices, etc.). Furthermore, validation of the eight clusters of behaviours through an in-depth qualitative inquiry of student users and stakeholders (university policymakers) can increase insights as to the implementation barriers which otherwise may not be captured within quantitative data collection, possibly due to the sensitive nature of the topic of drug-use as a research area. Finally, reporting of any innovations (e.g. personalization algorithms, computational models; e.g. [84]), resulting from turning the identified BCTs concepts into digital and interactive modules, can increase knowledge base for behavioural change interventions.

## Conclusions

In conclusion, this research provides an approach to applying the BCW approach to intervention development that draws upon primary and secondary data sources. The findings from the synthesis analysis enabled the identification of targeted problematic behaviours related to drug use. Increasing students’ mindful awareness in relation to drug-use decision making and promoting alternatives to drug-use activities indicate foci for the implementation of harm reduction strategies for higher education students’ drug-use. These can be delivered through a combination of the eight identified sources of drug-use behaviours. Researchers and implementation scientists can use the presented conceptual map to develop and design interventions and public policy strategies that can be sensitive to mitigate the harms resulting from the use of drugs within the context of higher education.

## Supplementary Information

**Additional file 1.** Main results from the Delphi type exercise, samples from the Pattern-based Grounded Discourse Analysis, and identification of MyUSE BCTs using the APEASE criteria.

## Data Availability

Not applicable.
