# Supplementary material for: Reducing drug-use harms among higher education students: MyUSE contextual-behaviour change digital intervention development using the Behaviour Change Wheel
Source: Harm Reduct J. 2021 May 20;18:56. doi: 10.1186/s12954-021-00491-7 (PMC8136195; doi:10.1186/s12954-021-00491-7)
Supplement: Supplementary file 1 — Additional file 1. Main results from the Delphi type exercise, samples from the Pattern-based Grounded Discourse Analysis, and identification of MyUSE BCTs using the APEASE criteria. [file 12954_2021_491_MOESM1_ESM.docx]

**Additional File 1:** Main results from the Delphi type exercise, samples from the Pattern-based Grounded Discourse Analysis, and identification of MyUSE BCTs using the APEASE criteria.

**Additional Table A1:** Advisory Group responses’ in the Delphi-type exercise- round one: Long List

| Item | Potential relevant to substance use behaviours (item’s description) | Round 1 (n=6) |  |  |  |  |
| --- | --- | --- | --- | --- | --- | --- |
|  |  |  | Mean (SD) | % of agreement^1^ | % of efficacy^2^ | Decision |
|  | **Domain A** | | | | | |
| A1 | Users to compare their own use with the norm (descriptive norm correction; e.g., % of students with higher use than their own) | Impact | 3.57 (0.54) | 57 |  | **approve** |
|  |  | Likelihood | 3.29 (0.49) | 28 |  |  |
|  |  | Spill over | 3.14 (1.01) | 28 |  |  |
|  |  | Measurement | 2.71 (1.70) | 43 |  |  |
|  |  | Efficacy | 1.71 (0.76) |  | 86 |  |
| A2 | Users to reflect on their close friends’ perception about their own use or abstinence (injunctive norm correction) | Impact | 3.87 (0.69) | 71 |  | **approve** |
|  |  | Likelihood | 3.71 (0.76) | 57 |  |  |
|  |  | Spill over | 3.29 (0.95) | 28 |  |  |
|  |  | Measurement | 2.57 (1.72) | 28 |  |  |
|  |  | Efficacy | 1.43 (0.54) |  | 100 |  |
| A3 | Users to complete a frequency of drug use and get a personalized feedback on their own pattern of use. | Impact | 4.0 (0.58) | 85 |  | **approve** |
|  |  | Likelihood | 3.43 (0.79) | 28 |  |  |
|  |  | Spill over | 2.86 (0.90) | 29 |  |  |
|  |  | Measurement | 4.0 (1.16) | 72 |  |  |
|  |  | Efficacy | 1.57 (0.54) |  | 100 |  |
| A4 | Users to reflect on their personal motivation for use (which are they? And why they take drugs? What prompt them to take drugs? Why they decide taking them?). | Impact | 4.14 (0.90) | 72 |  | **approve** |
|  |  | Likelihood | 3.71 (0.76) | 57 |  |  |
|  |  | Spill over | 3.14 (0.90) | 43 |  |  |
|  |  | Measurement | 2.29 (0.76) | 0 |  |  |
|  |  | Efficacy | 1.71 (0.76) | 72 | 86 |  |
| A5 | Users to find out their own primary motives for using drugs listing some form of personal enhancements they feel taking when consuming drugs (underlying needs that drive the decision to take drugs) | Impact | 3.71 (0.96) | 71 |  | **approve** |
|  |  | Likelihood | 3.29 (1.11) | 43 |  |  |
|  |  | Spill over | 3.29 (0.95) | 28 |  |  |
|  |  | Measurement | 3.0 (1.41) | 29 |  |  |
|  |  | Efficacy | 1.71 (1.41) |  | 71 |  |
| A6 | Users to understand the risks associated with experimental use. | Impact | 2.57 (1.27) | 29 |  | reject |
|  |  | Likelihood | 2.29 (1.38) | 14 |  |  |
|  |  | Spill over | 2.57 (0.98) | 14 |  |  |
|  |  | Measurement | 3.0 (1.0) | 14 |  |  |
|  |  | Efficacy | 2.14 (0.69) |  | 71 |  |
| A7 | Users to understand their motives of experimenting drugs in their context (when they need to engage in this behavior: context and thoughts they have). | Impact | 4.14 (0.69) | 86 |  | **approve** |
|  |  | Likelihood | 3.43 (0.98) | 43 |  |  |
|  |  | Spill over | 2.86 (0.90) | 14 |  |  |
|  |  | Measurement | 2.57 (1.51) | 14 |  |  |
|  |  | Efficacy | 1.86 (0.38) |  | 100 |  |
| A8 | User to become aware of their own negative consequences resulting from drug use. | Impact | 3.57 (1.13) | 43 |  | **approve** |
|  |  | Likelihood | 3.0 (1.15) | 43 |  |  |
|  |  | Spill over | 2.86 (1.35) | 29 |  |  |
|  |  | Measurement | 2.43 (1.51) | 29 |  |  |
|  |  | Efficacy | 1.86 (0.69) |  | 86 |  |
| A9 | Users to consider the cost-benefit analysis of their behavior (what they loose and gain). | Impact | 3.57 (1.27) | 58 |  | **approve** |
|  |  | Likelihood | 2.71 (1.25) | 29 |  |  |
|  |  | Spill over | 2.57 (1.61) | 43 |  |  |
|  |  | Measurement | 3.14 (1.57) | 43 | 99 |  |
|  |  | Efficacy | 1.43 (0.54) |  |  |  |
| A10 | Users to reflect on the possible negative consequences of using drugs on academic, athletic and social performance. | Impact | 4.14 (0.69) | 86 |  | **approve** |
|  |  | Likelihood | 3.29 (0.76) | 43 |  |  |
|  |  | Spill over | 3.57 (0.98) | 57 |  |  |
|  |  | Measurement | 3.14 (1.07) | 28 |  |  |
|  |  | Efficacy | 1.29 (0.49) |  | 100 |  |
| A11 | Users to reflect on their own choices (how taking drugs lead them toward vs. away value-based actions). | Impact | 3.71 (1.11) | 58 |  | **approve** |
|  |  | Likelihood | 3.71 (0.95) | 71 |  |  |
|  |  | Spill over | 3.57 (1.27) | 58 |  |  |
|  |  | Measurement | 2.14 (1.07) | 0 |  |  |
|  |  | Efficacy | 1.86 (0.69) |  | 86 |  |
| A12 | Users to reflect on scenarios of not using drugs or think of alternatives than drug use activities in their context (e.g., a night out without drugs.. what will happen?). | Impact | 3.0 (1.16) | 28 |  | **approve** |
|  |  | Likelihood | 2.71 (1.50) | 28 |  |  |
|  |  | Spill over | 2.43 (1.62) | 28 |  |  |
|  |  | Measurement | 2.14 (1.07) | 0 |  |  |
|  |  | Efficacy | 2.0 (1.0) |  | 86 |  |
| A13 | Users to assess annual expenses of substance use. | Impact | 2.86 (1.35) | 28 |  | reject |
|  |  | Likelihood | 2.57 (1.40) | 43 |  |  |
|  |  | Spill over | 2.0 (1.53) | 29 |  |  |
|  |  | Measurement | 3.57 (1.40) | 58 |  |  |
|  |  | Efficacy | 1.86 (1.22) |  | 71 |  |
| A14 | Users to consider whether the decide taking drugs under the influence of others or the pressure of being excluded by others. | Impact | 3.71 (1.11) | 58 |  | reject |
|  |  | Likelihood | 3.14 (1.07) | 28 |  |  |
|  |  | Spill over | 3.29 (0.95) | 28 |  |  |
|  |  | Measurement | 2.14 (1.07) | 14 |  |  |
|  |  | Efficacy | 2.29 (0.95) |  | 43 |  |
| A15 | Users to reflect on occasions in which drug use led them to do something embarrassing or felt ashamed/ guilt. | Impact | 3.86 (1.07) | 72 |  | **approve** |
|  |  | Likelihood | 3.57 (0.98) | 57 |  |  |
|  |  | Spill over | 3.14 (1.68) | 57 |  |  |
|  |  | Measurement | 2.86 (1.47) | 43 |  |  |
|  |  | Efficacy | 1.43 (0.54) |  | 100 |  |
| A16 | Users to reflect on occasions that drug use led to poor academic performance. | Impact | 3.86 (0.90) | 85 |  | **approve** |
|  |  | Likelihood | 3.86 (0.90) | 85 |  |  |
|  |  | Spill over | 2.86 (1.86) | 57 |  |  |
|  |  | Measurement | 3.14 (1.68) | 57 |  |  |
|  |  | Efficacy | 2.0 (0.82) |  | 72 |  |
| A17 | Users to recognize consistent and prolonged negative effects resulting from drug use across several aspects of their lives, as opposed to one single and predominant one. | Impact | 3.57 (0.79) | 43 |  | **approve** |
|  |  | Likelihood | 3.29 (1.38) | 57 |  |  |
|  |  | Spill over | 3.0 (1.41) | 43 |  |  |
|  |  | Measurement | 3.14 (1.07) | 28 |  |  |
|  |  | Efficacy | 2.0 (0.58) |  | 85 |  |
| A18 | Users to work on the perceived pros and cons of experimenting with drugs (weight pros and cons). | Impact | 2.29 (0.95) | 14 |  | reject |
|  |  | Likelihood | 2.57 (1.62) | 43 |  |  |
|  |  | Spill over | 1.71 (1.25) | 0 |  |  |
|  |  | Measurement | 2.86 (1.34) | 28 |  |  |
|  |  | Efficacy | 2.14 (0.69) |  | 71 |  |
| A19 | Users to be reinforced and get advice from counselling services on how to reduce the use. | Impact | 2.71 (1.38) | 43 |  | reject |
|  |  | Likelihood | 2.14 (1.35) | 14 |  |  |
|  |  | Spill over | 1.71 (1.70) | 29 |  |  |
|  |  | Measurement | 3.14 (1.07) | 28 |  |  |
|  |  | Efficacy |  |  | 43 |  |
| A20 | Users to increase awareness of what the drug use offer to them in the context of using (a prompt item). | Impact | 3.86 (1.07) | 72 |  | **approve** |
|  |  | Likelihood | 3.43 (1.13) | 57 |  |  |
|  |  | Spill over | 2.29 (1.50) | 29 |  |  |
|  |  | Measurement | 3.14 (1.22) | 43 |  |  |
|  |  | Efficacy | 2.29 (1.11) |  | 58 |  |
| A21 | Users to conduct a behavioural analysis (ABCs) of their behaviours based on the antecedents and consequences of using drugs in their context (a typical night out story and the negative consequences the day after). | Impact | 3.57 (1.13) | 43 |  | reject |
|  |  | Likelihood | 3.29 (0.95) | 28 |  |  |
|  |  | Spill over | 2.43 (1.13) | 0 |  |  |
|  |  | Measurement | 2.57 (1.13) | 29 |  |  |
|  |  | Efficacy | 2.29 (0.76) |  | 57 |  |
| A22 | Users to recognize their priorities fin their lives, based on the criteria of how important the drugs are and how they make them more caring human-being and help them in their personal development. | Impact | 3.71 (0.95) | 43 |  | reject |
|  |  | Likelihood | 3.29 (1.25) | 43 |  |  |
|  |  | Spill over | 3.14 (1.57) | 43 |  |  |
|  |  | Measurement | 2.71 (0.95) | 14 |  |  |
|  |  | Efficacy | 1.86 (0.90) |  | 72 |  |
| A23 | Users to reflect on “real” friends being there for them vs. friend who only be together to do drugs. | Impact | 2.71 (0.76) | 14 |  | reject |
|  |  | Likelihood | 2.86 (1.07) | 14 |  |  |
|  |  | Spill over | 2.14 (1.07) | 0 |  |  |
|  |  | Measurement | 2.29 (1.25) | 14 |  |  |
|  |  | Efficacy | 2.71 (1.11) |  | 43 |  |
| A24 | Users to record time spent on tv, games (play-station, x-box, etc.) and social media use while using drugs and rate their importance & impact on their lives. | Impact | 2.57 (0.78) | 0 |  | reject |
|  |  | Likelihood | 2.43 (1.51) | 29 |  |  |
|  |  | Spill over | 2.29 (1.60) | 14 |  |  |
|  |  | Measurement | 3.57 (1.40) | 58 |  |  |
|  |  | Efficacy | 3.14 (1.07) |  | 14 |  |
| A25 | Users to recall a night out that involved drug use and this resulted in doing something embarrassing which they regretted the other day. | Impact | 3.57 (0.79) | 43 |  | **approve** |
|  |  | Likelihood | 3.14 (1.46) | 57 |  |  |
|  |  | Spill over | 3.14 (1.86) | 58 |  |  |
|  |  | Measurement | 3.14 (1.07) | 28 |  |  |
|  |  | Efficacy | 1.71 (0.76) |  | 86 |  |
| A26 | Users to reflect on a factitious story of a UCC student (a narrative frame or a drawing animation) whose all his/her fears went true because of drug use (see Jane’s fears). | Impact | 3.14 (0.90) | 14 |  | reject |
|  |  | Likelihood | 2.71 (1.38) | 28 |  |  |
|  |  | Spill over | 2.71 (1.80) | 29 |  |  |
|  |  | Measurement | 2.57 (1.62) | 28 |  |  |
|  |  | Efficacy | 2.57 (0.54) |  | 43 |  |
| A27 | Users to become aware of the reasons they compete with others in terms of appearance, marks, social popularity and attractiveness- low self-esteem? | Impact | 2.29 (0.76) | 0 |  | reject |
|  |  | Likelihood | 2.43 (0.54) | 0 |  |  |
|  |  | Spill over | 2.29 (1.25) | 14 |  |  |
|  |  | Measurement | 2.29 (0.76) | 0 |  |  |
|  |  | Efficacy | 3.14 (0.38) |  | 0 |  |
| A28 | Users to reflect on their own use through a track diary device. | Impact | 2.86 (0.69) | 14 |  | **approve** |
|  |  | Likelihood | 3.0 (1.0) | 29 |  |  |
|  |  | Spill over | 2.71 (1.25) | 14 |  |  |
|  |  | Measurement | 3.71 (1.80) | 72 |  |  |
|  |  | Efficacy | 2.71 (0.49) |  | 29 |  |
|  | **Domain B** | | | | | |
| B1 | Users to get advice on learning emotional regulation skills to ease unwanted emotions (e.g., performance anxiety, etc.). | Impact | 2.71 (1.25) | 43 |  | **approve** |
|  |  | Likelihood | 3.0 (1.29) | 28 |  |  |
|  |  | Spill over | 3.29 (1.70) | 71 |  |  |
|  |  | Measurement | 2.29 (1.60) | 14 |  |  |
|  |  | Efficacy | 2.57 (0.54) |  | 43 |  |
| B2 | Users to learn mindfulness practices to be aware of their own behaviours on a daily basis. | Impact | 3.0 (1.41) | 43 |  | reject |
|  |  | Likelihood | 2.71 (1.25) | 14 |  |  |
|  |  | Spill over | 3.29 (1.60) | 57 |  |  |
|  |  | Measurement | 2.14 (1.22) | 0 |  |  |
|  |  | Efficacy | 2.29 (1.11) |  | 58 |  |
| B3 | Users to get advice on alternative activities | Impact | 2.86 (1.46) | 28 |  | reject |
|  |  | Likelihood | 3.14 (1.35) | 43 |  |  |
|  |  | Spill over | 3.29 (1.60) | 57 |  |  |
|  |  | Measurement | 2.57 (0.98) | 14 |  |  |
|  |  | Efficacy | 2.29 (0.95) |  | 43 |  |
| B4 | Users to learn social skills training, particularly in becoming more assertive to say no to drug use. | Impact | 3.57 (0.54) | 57 |  | **approve** |
|  |  | Likelihood | 3.43 (0.79) | 28 |  |  |
|  |  | Spill over | 3.57 (1.62) | 85 |  |  |
|  |  | Measurement | 2.57 (0.79) | 14 |  |  |
|  |  | Efficacy | 2.43 (0.79) |  | 71 |  |
| B5 | Users to learn self- time management skill training to cope with daily tasks. | Impact | 2.14 (0.90) | 0 |  | reject |
|  |  | Likelihood | 2.86 (1.35) | 28 |  |  |
|  |  | Spill over | 2.57 (1.40 | 29 |  |  |
|  |  | Measurement | 3.43 (0.98) | 43 |  |  |
|  |  | Efficacy | 2.71 (0.49) |  | 29 |  |
| B6 | Users to learn skills to reduce compulsiveness (automatic decision to take drugs). | Impact | 3.71 (0.95) | 71 |  | **approve** |
|  |  | Likelihood | 3.43 (1.27) | 57 |  |  |
|  |  | Spill over | 3.57 (1.72) | 72 |  |  |
|  |  | Measurement | 2.71 (0.95) | 29 |  |  |
|  |  | Efficacy | 2.14 (1.22) |  | 57 |  |
| B7 | Users to identify activities that motivate them (fighting laziness). | Impact | 3.0 (1.29) | 28 |  | reject |
|  |  | Likelihood | 2.86 (1.46) | 28 |  |  |
|  |  | Spill over | 3.14 (1.77) | 43 |  |  |
|  |  | Measurement | 2.86 (1.22) | 28 |  |  |
|  |  | Efficacy | 2.43 (0.78) |  | 43 |  |
| B8 | Users to learn skills to combat negative automatic thoughts. | Impact | 2.71 (1.38) | 43 |  | **approve** |
|  |  | Likelihood | 3.0 (1.16) | 43 |  |  |
|  |  | Spill over | 3.29 (1.70) | 71 |  |  |
|  |  | Measurement | 2.86 (0.90) | 29 |  |  |
|  |  | Efficacy | 2.43 (0.98) |  | 57 |  |
| B9 | Users to record time spent with friends, family and themselves and reflect on the time spent with significant others (ideal vs. reality). | Impact | 2.14 (1.22) | 14 |  | reject |
|  |  | Likelihood | 2.43 (0.78) | 0 |  |  |
|  |  | Spill over | 2.29 (1.25) | 14 |  |  |
|  |  | Measurement | 3.0 (1.16) | 43 |  |  |
|  |  | Efficacy | 3.0 (0.82) |  | 29 |  |
|  | **Domain C** | | | | | |
| C1 | Users to identify personal values and behavioural goals that lead to committed actions (prompt item to reinforce desired behaviours fulfilling personal enhancements.). | Impact | 3.57 (0.79) | 43 |  | reject |
|  |  | Likelihood | 3.14 (1.07) | 28 |  |  |
|  |  | Spill over | 3.14 (1.57) | 43 |  |  |
|  |  | Measurement | 2.86 (1.35) | 28 |  |  |
|  |  | Efficacy | 2.0 (0.82) |  | 72 |  |
| C2 | Users to get advice on how to overcome personal challenges in relation to college attendance, maintenance of a heathy eating habit (daily), leaving friends (spending time on his own), and being independent (paying his/her bills, completing assignments, etc.) | Impact | 3.0 (0.82) | 29 |  | **approve** |
|  |  | Likelihood | 2.86 (0.90) | 29 |  |  |
|  |  | Spill over | 2.86 (1.35) | 29 |  |  |
|  |  | Measurement | 2.71 (0.95) | 29 |  |  |
|  |  | Efficacy | 1.86 (0.38) |  | 100 |  |
| C3 | Users to learn and practice self-compassion skills (being themselves, not pretending being others, try fitting in, fighting shame & embracement). | Impact | 3.14 (0.69) | 29 |  | reject |
|  |  | Likelihood | 2.57 (0.54) | 0 |  |  |
|  |  | Spill over | 3.0 (1.41) | 43 |  |  |
|  |  | Measurement | 2.57 (1.40) | 14 |  |  |
|  |  | Efficacy | 2.43 (0.98) | 29 | 57 |  |
| C4 | Users to build on activities that promote a continue self of self with an emphasis in mindful observing their thoughts/emotions (observer self) rather than getting caught up by them (conceptualized self). | Impact | 3.43 (0.79) | 57 |  | reject |
|  |  | Likelihood | 2.71 (0.49) | 0 |  |  |
|  |  | Spill over | 3.29 (1.60) | 57 |  |  |
|  |  | Measurement | 2.71 (1.11) | 14 |  |  |
|  |  | Efficacy | 2.29 (1.11) |  | 58 |  |
|  | **Domain D** | | | | | |
| D1 | Users to learn how to help others who misuse drugs | Impact | 3.0 (0.89) | 33 |  | reject |
|  |  | Likelihood | 2.67 (1.97) | 50 |  |  |
|  |  | Spill over | 1.83 (1.60) | 17 |  |  |
|  |  | Measurement | 3.17 (0.98) | 50 |  |  |
|  |  | Efficacy | 2.67 (1.03) |  | 34 |  |
| D2 | Users to be informed of the best way to intervene if they feel concerned about a friend/ peer, etc. (attempts to rectify social norms). | Impact | 3.50 (0.84) | 67 |  | reject |
|  |  | Likelihood | 3.33 (1.75) | 67 |  |  |
|  |  | Spill over | 3.33 (1.21) | 50 |  |  |
|  |  | Measurement | 3.33 (1.21) | 67 |  |  |
|  |  | Efficacy | 2.33 (1.21) |  | 50 |  |
| D3 | Users to dispute a common misperception that illicit substance use and misuse will help them combat some of the negative consequences of college life and help them to manage their time better. | Impact | 3.0 (0.63) | 17 |  | reject |
|  |  | Likelihood | 2.83 (1.17) | 17 |  |  |
|  |  | Spill over | 3.33 (1.03) | 34 |  |  |
|  |  | Measurement | 2.83 (1.33) | 50 |  |  |
|  |  | Efficacy | 2.33 (0.52) |  | 67 |  |
| D4 | Users to examine the perceived effect (e.g., in academic, social and athletic performance) vs. the actual effects of substance use- find the discrepancies. | Impact | 3.33 (0.82) | 50 |  | reject |
|  |  | Likelihood | 3.50 (0.84) | 67 |  |  |
|  |  | Spill over | 3.0 (1.27) | 50 |  |  |
|  |  | Measurement | 3.33 (0.82) | 50 |  |  |
|  |  | Efficacy | 2.17 (1.17) |  | 66 |  |
| D5 | Users to have access to info and material on treatment options across the community | Impact | 2.60 (1.14) | 20 |  | reject |
|  |  | Likelihood | 2.00 (1.23) | 20 |  |  |
|  |  | Spill over | 2.80 (1.30) | 40 |  |  |
|  |  | Measurement | 2.60 (1.34) | 40 |  |  |
|  |  | Efficacy | 3.00 (1.23) |  | 20 |  |
|  | **Domain E** | | | | | |
| E1 | Users to get motivated to increase communication with family | Impact | 2.0 (0.89) | 0 |  | reject |
|  |  | Likelihood | 2.50 (1.05) | 17 |  |  |
|  |  | Spill over | 2.83 (1.47) | 34 |  |  |
|  |  | Measurement | 3.0 (0.89) | 33 |  |  |
|  |  | Efficacy | 2.67 (0.82) |  | 17 |  |
| E2 | Users to seek help from friends who believe they can encourage them in the process of reducing the use | Impact | 2.50 (1.05) | 17 |  | reject |
|  |  | Likelihood | 2.67 (0.82) | 17 |  |  |
|  |  | Spill over | 3.0 (1.27) | 34 |  |  |
|  |  | Measurement | 2.83 (0.75) | 17 |  |  |
|  |  | Efficacy | 3.17 (0.75) |  | 17 |  |
| E3 | Users to designate others (e.g., friends) to increase pressure for reducing the use | Impact | 1.83 (0.75) | 0 |  | reject |
|  |  | Likelihood | 2.33 (1.03) | 17 |  |  |
|  |  | Spill over | 2.50 (1.38) | 17 |  |  |
|  |  | Measurement | 2.67 (0.82) | 17 |  |  |
|  |  | Efficacy | 3.17 (0.41) |  | 0 |  |
| E4 | Users to recognize the widespread acceptance of the negative consequences of drug use among those who misuse. | Impact | 2.17 (0.75) | 0 |  | reject |
|  |  | Likelihood | 2.50 (1.05) | 17 |  |  |
|  |  | Spill over | 3.17 (1.17) | 34 |  |  |
|  |  | Measurement | 2.50 (0.55) | 0 |  |  |
|  |  | Efficacy | 2.67 (0.82) |  | 50 |  |
| E5 | Users to hear others expressing concerns about their drug use (even fantasazing others how they see them in relation to drugs). | Impact | 3.17 (0.98) | 17 |  | **approve** |
|  |  | Likelihood | 2.50 (1.05) | 17 |  |  |
|  |  | Spill over | 3.33 (1.51) | 50 |  |  |
|  |  | Measurement | 2.50 (0.84) | 17 |  |  |
|  |  | Efficacy | 2.00 (0.63) |  | 84 |  |
| E6 | Users to reflect on how others see them, particularly partners, family members and close friends. | Impact | 3.83 (1.17) | 66 |  | **approve** |
|  |  | Likelihood | 3.33 (1.37) | 50 |  |  |
|  |  | Spill over | 4.17 (0.75) | 83 |  |  |
|  |  | Measurement | 2.83 (0.98) | 33 |  |  |
|  |  | Efficacy | 1.83 (1.17) |  | 83 |  |

Note 1: Data are expressed as percentage of responses; % Agreement = a sum score of those who ticked the last two responses, very and extremely in the APEASE criteria.

Note 2: efficacy followed a four-Likert type scale and assessed individually; % efficacy = those who ticked the last two responses, very and quite efficacy (>80%). Items scored >80% in the efficacy were automatically included in the shorter-list irrespective of the APEASE score.

**Additional** **Table A2**: Advisory Group’s responses in the Delphi-type exercise- round two: Short List

| Item | Potential relevant to substance use behaviours (item’s description) | | Round 2 (n=6) |  |  |  |  |  | Decision |
| --- | --- | --- | --- | --- | --- | --- | --- | --- | --- |
|  |  | |  | Mean (SD) | Median | % of agreement^1^ | Mean median^2^ | Mean % of agreement^3^ |  |
|  | | **Domain A** | | | | | | | |
| A1 | **Users to compare their own use with the norm (descriptive norm correction; e.g., % of students with higher use than their own)** | | Impact | 4.29 (0.75) | 4 | 85.8 | 4 | 82.2 | **Approve^4^** |
|  |  |  | Likelihood | 4.29 (0.49) | 4 | 100 |  |  |  |
|  |  |  | Spill over | 3.57 (0.79) | 4 | 71.4 |  |  |  |
|  |  |  | Measurement | 3.71 (1.25) | 4 | 71.5 |  |  |  |
|  |  |  | Efficacy | 2.43 (0.53) | 2 |  |  |  |  |
| A2 | **Users to reflect on their close friends’ perception about their own use or abstinence (injunctive norm correction)** | | Impact | 4.71 (0.49) | 5 | 100 | 4 | 78.8 | **approve** |
|  |  |  | Likelihood | 4.29 (0.49) | 4 | 100 |  |  |  |
|  |  |  | Spill over | 4.29 (0.49) | 4 | 100 |  |  |  |
|  |  |  | Measurement | 2.71 (0.76) | 3 | 14.3 |  |  |  |
|  |  |  | Efficacy | 2.43 (0.53) | 2 |  |  |  |  |
| A3 | Users to complete a frequency of drug use and get a personalized feedback on their own pattern of use. | | Impact | 3.86 (0.90) | 4 | 58.4 | 3.75 | 72 | reject |
|  |  |  | Likelihood | 4.00 (5.76) | 4 | 85.7 |  |  |  |
|  |  |  | Spill over | 3.29 (0.76) | 3 | 42.9 |  |  |  |
|  |  |  | Measurement | 4.43 (0.53) | 4 | 100 |  |  |  |
|  |  |  | Efficacy | 2.29 (0.49) | 2 |  |  |  |  |
| A4 | Users to reflect on their personal motivation for use (which are they? And why they take drugs? What prompt them to take drugs? Why they decide taking them?). | | Impact | 4.29 (0.95) | 5 | 71.4 | 4.25 | 64 | reject |
|  |  |  | Likelihood | 3.71 (0.76) | 4 | 57.2 |  |  |  |
|  |  |  | Spill over | 3.71 (0.76) | 4 | 57.2 |  |  |  |
|  |  |  | Measurement | 3.86 (1.07) | 4 | 71.5 |  |  |  |
|  |  |  | Efficacy | 2.57 (0.79) | 2 |  |  |  |  |
| A5 | **Users to find out personally relevant primary motives for use, listing the personal enhancements drug use fulfil to them** | | Impact | 4.71 (0.49) | 5 | 100 |  |  |  |
|  |  |  | Likelihood | 3.86 (0.90) | 4 | 85.7 | 4.25 | 78 | **approve** |
|  |  |  | Spill over | 4.00 (5.77) | 4 | 85.7 |  |  |  |
|  |  |  | Measurement | 3.43 (0.97) | 3 | 42.9 |  |  |  |
|  |  |  | Efficacy | 4.00 (0.58) | 3 |  |  |  |  |
| A8 | User to become aware of their own negative consequences resulting from drug use. | | Impact | 3.29 (1.38) | 3 | 85.8 | 3.75 | 64 | reject |
|  |  |  | Likelihood | 3.29 (1.25) | 4 | 57.2 |  |  |  |
|  |  |  | Spill over | 3.43 (1.62) | 3 | 42.9 |  |  |  |
|  |  |  | Measurement | 3.00 (1.29) | 3 | 71.5 |  |  |  |
|  |  |  | Efficacy | 2.29 (0.76) | 2 |  |  |  |  |
| A9 | Users to consider the cost-benefit analysis of their behavior (what they loose and gain). | | Impact | 4.29 (0.76) | 4 | 85.8 | 3.50 | 57 | reject |
|  |  |  | Likelihood | 3.57 (0.98) | 4 | 42.9 |  |  |  |
|  |  |  | Spill over | 3.43 (0.98) | 3 | 57.2 |  |  |  |
|  |  |  | Measurement | 4.00 (1.15) | 4 | 42.9 |  |  |  |
|  |  |  | Efficacy | 2.71 (0.49) | 3 |  |  |  |  |
| A10 | **Users to reflect on the possible negative consequences of using drugs on academic, athletic and social performance.** | | Impact | 4.29 (0.76) | 4 | 100 | 4 | 75 | **approve** |
|  |  |  | Likelihood | 3.43 (1.27) | 3 | 85.7 |  |  |  |
|  |  |  | Spill over | 3.43 (1.27) | 4 | 71.5 |  |  |  |
|  |  |  | Measurement | 3.29 (1.50) | 3 | 42.9 |  |  |  |
|  |  |  | Efficacy | 2.29 (0.76) | 2 |  |  |  |  |
| A15 | Users to reflect on occasions in which drug use led them to do something embarrassing or felt ashamed/ guilt. | | Impact | 4.57 (0.53) | 5 | 71.4 | 3.75 | 43 | reject |
|  |  |  | Likelihood | 4.14 (0.69) | 4 | 57.1 |  |  |  |
|  |  |  | Spill over | 4.00 (1.15) | 4 | 42.9 |  |  |  |
|  |  |  | Measurement | 3.43 (0.98) | 3 | 14.3 |  |  |  |
|  |  |  | Efficacy | 2.57 (0.53) | 3 |  |  |  |  |
| A25 | Users to recall a night out that involved drug use and this resulted in doing something embarrassing which they regretted the other day. | | Impact | 4.00 (1.09) | 4 | 71.5 | 3.75 | 57 | reject |
|  |  |  | Likelihood | 3.50 (1.22) | 3 | 71.4 |  |  |  |
|  |  |  | Spill over | 3.17 (1.17) | 3 | 57.2 |  |  |  |
|  |  |  | Measurement | 2.67 (1.37) | 2.5 | 28.6 |  |  |  |
|  |  |  | Efficacy | 2.00 (0.89) | 2 |  |  |  |  |
|  | | **Domain B** | | | | | | | |
| B6 | Users to learn skills to reduce compulsiveness (automatic decision to take drugs). | | Impact | 4.00 (0.82) | 4 | 57.2 | 3.50 | 47 | reject |
|  |  |  | Likelihood | 3.14 (1.35) | 3 | 42.9 |  |  |  |
|  |  |  | Spill over | 4.14 (1.10) | 4 | 85.8 |  |  |  |
|  |  |  | Measurement | 2.71 (0.49) | 3 | 0 |  |  |  |
|  |  |  | Efficacy | 2.43 (0.53) | 2 |  |  |  |  |
|  | | **Domain E** | | | | | | | |
| E6 | Users to reflect on how others see them, particularly partners, family members and close friends. | | Impact | 3.83 (1.17) | 5 | 85.7 | 3.87 | 53 | reject |
|  |  |  | Likelihood | 3.33 (1.37) | 4.5 | 71.5 |  |  |  |
|  |  |  | Spill over | 4.17 (0.75) | 4 | 42.9 |  |  |  |
|  |  |  | Measurement | 2.83 (0.98) | 2 | 14.3 |  |  |  |
|  |  |  | Efficacy | 1.83 (1.17) | 2 |  |  |  |  |

Note1: Data are expressed as percentage of responses; % Agreement = a sum score of those who ticked the last two responses, very and extremely in the APEASE criteria; Note 2: Mean median scores for the four APEASE criteria (only those reached the cut off > 3.25 (Hasson et al., 2000); Note 3:Mean % score agreement of the APEASE criteria (% of those who ticked very and extremely/ 4) that reached the >70% agreement score; Note 4: the four final approved behaviors to target are highlighted.

**Additional Table A3**: Examples from coding data in each of the four steps of the Pattern-based Grounded Discourse Analysis

| Steps in Discourse Analysis | Brief Description | Example from Coding Analysis |
| --- | --- | --- |
| **Step 1: Open Coding** | **Example 1:** Selective item selected from the survey and analysed, firstly, quantitatively, and then a qualitative descriptors was added to explain an aspect of the targeting behaviours through a COM-B lens | **RM_2**   \| Item \| In your opinion, what effect does drug use have on student life? \| \| --- \| --- \| \| Item’s COM-B coded \| RM_2 \| \| Item’s SPSS coded \| S3_Q1b \| \| Regrouping variable \| (1=1) (2=1) (3=3) (4=4) (5=4) \| \| Direction of COM-B component (mean of 1 and 2) \| Increase MR on the effect of drug on their lives. \| \| Brief statement (what does this mean?) \| The majority of students generally holds a negative belief about the effects of drugs on student life. That means that by increasing reflective motivation it also expected to increase the targeted behavior to change. \|  \| N \| Mean (SD) \| Range \| Scale’s responses (valid percentages; %) \| \| \| \| \| \| \| \| --- \| --- \| --- \| --- \| --- \| --- \| --- \| --- \| --- \| --- \| \| Extremely negative effect (1)  % (n) \| Somewhat negative effect (2) \| Neither negative or positive effect (3) \| Somewhat positive effect (4) \| Extremely positive effect  (5) \| % (n); perceived drug as having negative effect scores responses 1+2 \| \| \| 967 \| 2.30 \| 1-5 \| 24% (231) \| 53% (508) \| 17% (164) \| 9% (56) \| 0.7 (7) \| 38% (739) \|  \|  \| S3_Q1b_Reg \|  \|  \|  \|  \|  \| \| --- \| --- \| --- \| --- \| --- \| --- \| \|  \|  \| Frequency \| Percent \| Valid Percent \| Cumulative Percent \| \| Valid \| Negative effect of drugs to student life \| 740 \| 65.0 \| 77.1 \| 77.1 \| \|  \| Positive effect of drugs to student life \| 220 \| 19.3 \| 22.9 \| 100.0 \| \|  \| Total \| 960 \| 84.4 \| 100.0 \|  \| \| Missing \| System \| 178 \| 15.6 \|  \|  \| \| Total \|  \| 1138 \| 100.0 \|  \|  \|   **RM_8**   \| Item \| Thinking about how, when and why you use drugs, do you think your use would **change** if you... \| \| --- \| --- \| \| Item’s COM-B coded \| RM_8 \| \| Item’s SPSS coded \| S5b_5_GROUP_1 till S5b_5_GROUP_8 \| \| Direction of COM-B component \| Increase reflective motivation (intention) for students to start alternative to drug use activities. \| \| Brief statement (what does this mean?) \| Reflective motivation should be enhanced and in relation to alternative drug use activities. \|  \|  \|  \| Responses \|  \| Percentage of cases \| \| --- \| --- \| --- \| --- \| --- \| \|  \|  \| N \| Percentage \|  \| \|  \| {1, Found a different way to manage my emotions}... \| 70 \| 10.5 \| 31.4 \| \|  \| {1, Felt confident to say no}... \| 44 \| 6.6 \| 19.7 \| \|  \| {1, Felt like I was capable of making changes}... \| 55 \| 8.2 \| 24.7 \| \|  \| {1, Felt confident I would have a good time without using drugs}... \| 123 \| 18.4 (3) \| 55.2 \| \|  \| {1, Spent time doing other things}... \| 160 \| 24 (1) \| 71.7 \| \|  \| {1, Spent time with a different social group}... \| 125 \| 18.7 (2) \| 56.1 \| \|  \| {1, Knew more about the risks involved with drug use}... \| 62 \| 9.3 \| 27.8 \| \|  \| {1, Other (please state)}... \| 28 \| 4.2 \| 12.6 \| \| Total \|  \| 667 \| 100 \| 299.1 \| |
| **Step 2: Axial Coding** | **Example 1:** of coding when COM-B components are considered as central phenomena. Data are coded, deductively, assembling the qualitative descriptors in the COM-B components  **Example 2:** Deductive coding of data, assembling the qualitative descriptors, generated previously, in the COM-B components and then systematically relating these descriptors into TDF categories | \| **Central Phenomena (COM-B)** \| **Descriptors** \| **Saturation** \| **What needs to happen for the target behaviour to occur?** \| **Is there a need for a change?** \| **What needs to change?** \| \| --- \| --- \| --- \| --- \| --- \| --- \| \| ID: AM-4, AM-5, AM-6 \| \| \| \| \| \| \| **Motivation** \| Students drug use related behaviours are  contingency related with automatic desired outcomes (that is, taking drug will increase a desired behaviours; e.g., confidence, energy levels and decrease in unwanted ones; e.g., lower anxiety). This is habitually followed every time student engage in drug-related behaviours, but the expected outcomes in the long-turn become aversive (e.g., decrease in energy levels, in social interaction and confidence and increase in irritability, distress). \| Students’ expectations about positive effects resulting from substance use are misleading  Students take substances expecting increases in personally desired behavior changes  Students’ expectations about increases in personally desired behaviours from the use of drugs in the long-run, are not fulfilled \| 1. Students should learn to:  - brake automatic/habitual use - reflect on their own use and the need (desired effects) for taking drugs - understand their desires, drive states (motivation for use) in the context and decide mindfully - allocate time to decide consciously (without being driven by automatic reactions) by reflecting on their previous experiences (what are the previously experienced both immediate and in the long-run resulted from taking drugs?) \| 1. Change is needed as there are conflicted responses of the short and long-term consequences from drug-related behaviours (students do not recognize the reported negative effects of drug use) \| - Resolve misleading expectations about the expected outcomes drug use have on desired behaviours - Increase perception about the real effects’ drug use have on students’ personally desired behaviours \| \| 1. Students should become aware of their own long-term consequences from drug use (not respond automatically to emotional reactions, expected outcomes and desires) \| 1. Change is needed as students clearly report several areas of daily functioning being affected from the use of drugs. \| \| 1. Students should discover alternative reinforcers (value-based actions) to fulfil their desires \| 1. Change is needed as students report lack of recognizing alternatives for drug use \| \| ID: RM-3, RM-9, RM-10, RM-11 & RM-15 \| \| \| \| \| \| \|  \| Students hold specific beliefs/ evaluations about drug-related behaviours (perceived reasons) and consequences (reflect evaluations) that may increase motivation for abstaining. \| Cultivate mindful awareness of the perceived reasons for using and expected outcomes in students’ decision regarding any substance use related  behaviours \| Increase awareness and motivation for change (optimism for success), particularly in making students:   - aware of their social role and how drugs interfere with this role (how others see them) - increase a conscious decision to do something regarding drug use (implementation intention); - reflect on how drugs evoke concerns and certain distressing experiences (anxiety, depression, etc.) - increase reflective evaluations for the effects of substances in significant areas of students’ lives (effects in menta & physical capacity, financial impacts) - provide alternatives to drug use with specific action planning (goals- value committed actions); - increase and reinforce outcome expectancies resulting from new consequences (e.g., from value-based activities vs. drug ones) \| Change is needed , particularly there is a need for enhancing reflective motivation as student do not recognize the value of alternative value activities in the long-run \| - Mindful awareness of the reasons for use - Provide alternatives to drug use with specific action planning \| \| IDs: [CPh-1 & CPh-2] \| \| \| \| \| \| \| Capability \| Students present with insufficient knowledge, skills, and information on how to use harm-reduction practices.  However, they present with sufficient self-efficacy and optimism to stop using drugs. \| Students don’t know how to apply skills (measures) to reduce the harm-associated with substance use. \| 1. Increase skills/knowledge about appropriate measures that can reduce the risk associated with substances \| 1. Change needed as students report only moderate knowledge/ skills for measures that can reduce drug use. \| - Psychological capability should not be a COM-B target as students have the knowledge and mental capacity to protect themselves from the harm associated with substances. - Focus should be given in practical skills, and the knowledge on how to use harm-reduction practices \| \| 1. Increase optimism, self-confidence and self-efficacy that specific measures can be applied to reduce the risks associated with substances (expected outcomes) \| 1. No change needed as survey show students have enough confidence. \| \| 1. Increase self-efficacy and optimism so that students feel empowered that by using specific measures, they should be able to effectively reduce the risk associated with drugs should be able to effectively reduce the risk associated with drugs. \| 1. No change needed as survey show students hold this knowledge. \|  \| Qualitative Descriptors \| COM-B identified \| # of TDF Domains (n) \| Reasons for selecting the TDF \| \| --- \| --- \| --- \| --- \| \| Perceived **short-**term consequences in daily functioning from substance use \| Automatic motivation \| 8 \| - Identity= Students reported that drug use enhance their social identify roles (increase confidence and social interaction) - Goals= students take drugs to achieve a proximal goal (increase a rewarding behavior or decrease an unwanted experience) - Beliefs about consequences= increase in energy levels, social interaction, confidence, relaxation levels, and decrease in irritability & anxiety - Reinforcement= there are incentives for completing the behavior (they will achieve something; a decrease or increase in a targeted-behavior) - Emotions= taking drugs result in an expected emotional response (reduction in anxiety, irritability & increase in relaxation) - Enviro cont= expected outcomes are driven mostly by environmental triggering factors (interpersonal or contextual) - Cog= students take drugs as a coping skill in emotional responses or interpersonal reactions - Beh. Regulation =students take drugs to regulate an action \| \| Perceived reasons for stopping using substances \|  \| 6 \| - Id= student report reasons for stop using drugs in several social identity areas (academic, 36%; personal physical safety; 33% and academic roles; 36%) - Int= students report a conscious decision to perform a behavior (stop using drugs) and act in a certain (valued) way; e.g., 35% report concerns about academic impacts; and cognitive ones, 45%) - Goals= students present a mental representation of stop using drugs to achieve a certain short or long-term outcome (complete modules, being safe; 33%; not having legal problems; 36%) - Bel. Cons= students express beliefs about certain consequences that may occur as a result of drugs cessation (e.g., less cognitive and academic problems; 45 & 36%, respectively; less psychological burden; 60% and physical fatigue; 38%, etc.) - Reinf.= students increase the likelihood of drug use cessation (a response) if a contingency occuers between this response (stop using drugs) and a given automatic reflection (an anticipated pleasure; I will have better concentration, less academic problems, less psychological distress, and impact on my physical stamina and safety) - Em= students report that stop using drugs will evoke less negative emotional responses (anxiety, sleep, depression) or threats for legal consequences \| \| Change in drug use behaviours follows noticeable effects in functioning and aspects of college life \|  \| 8 \| - Id= 39% of students taking drugs. reported conflicts between their drug use and concerns about their professional identity - Int= 40% of students have reported a conscious decision to change substance use (perform a behavior or act in a certain way) to increase their daily functioning. - Bel exp= ~ 40% of students reported willingness to change their drug use (implementation intention) - Reinf= students reported at least 9 areas of daily functioning (> 30%) in which they noticed reduction in productivity that enacted behavioral changes in drug use patterns. - Em=59% of students reported that drug use evoked negative psychological reactions (depression, anxiety, etc.) to some extent that led them to change their pattern of use. - Social inf= 34% of students reported that their drug use resulted to some forms of social intrapersonal conflicts (e.g., isolation, judgement) that led to behavioural changes in drug use. Also, 38% of students taking drugs reported injunctive norm problems (how others perceive their drug use) that led to behavioural changes in drug use. - Environmental context: Students reported concerns resulting from drug use that made them to change their patterns in at least three areas of life/functioning (person x environmental interaction):  1. 30% concerns about legal implications 2. 48% expressed concerns for financial problems 3. 41% concerns about physical effects (fatigue, low energy, poor diet, etc.)  - Mem= 39% of students reported noticeable mental impairments (decision making, memory, etc.) resulted from drug use that made them change their pattern of use. \| |
| **Step 3: Selective Coding** | **Example:** Synthesis of qualitative descriptors (selective codes) for narrative developments statements describing TDF | \|  \| **TDF Domain** \| Selective qualitative descriptors \| Narrative statements \| \| --- \| --- \| --- \| --- \| \| *Reflective Motivation* \| **Beliefs about consequences:**  **Immediate fulfilment of students’ expected outcomes (e.g., increase in energy level, etc)- short lived BUT not in the long-run** \| - AM-4 +AM-5= students hold positive beliefs about expected outcomes (increase in energy levels, social interaction, confidence, relaxation levels, and decrease in irritability & anxiety). - RM-0= students expect to have fun with the use of substances. - RM-1= students express concern about their personal physical safety (54.6%); beliefs about their own self efficacy in daily life when using drugs. - RM-2= the majority of students belief that drug use can have a general (overall) negative effect to their life (77.1%) - RM-3= students’ reflective motivation to stop using drugs are related with expected negative outcomes in psychological (e.g., anxiety will increase, sleep will be disrupted, depression will follow euphoria, etc.), cognitive (perception will diminish), and academic (drugs will affect academic progress) areas of living - RM-4= student valued identity role is related with specific expected outcomes (expected from someone in HEI to do drugs) - RM-5= the outcome expectances from questioning future use are very low. - RM-7= students expect to use substances several hours before use (45%), but also several days (38%) - RM-8 = students report changing their current substance use, if the expected outcome of an alternative to substance use, activity fulfil a desired outcome (have fun; 55%). - RM-11= only ½ of the students hold beliefs (thoughts) for expected ways to reduce drug use (outcomes). - RM-14= students attribute the increase risk of taking substances during college to the freedom this context provides (58%) and the lack of negative consequences of these behaviours (61%) - CPh-1= students report moderate faith in using harm-reduction practices to address the consequences of drug use. - Cph-2= students report an expected outcome resulting from their behavior. - CPs_2= students report that if they avoid certain environments or friend who frequently use drugs, they can reach an ending goal. - S0-2= students report that the presence of other who take drugs may increase the likelihood to take drugs (32%) - S_03= students believe that being around other who attempt to reduce their drug use would result in decreasing drug use (72%). \| Use is often associated with students’ perceived utility in achieving some form of personal enhancement (outcome expectancies). In this regard, students perceived expectations in achieving some form of personal enhancement from use may facilitate future use. This occurs as a result of substances’ function to temporarily fulfil students’ perceived expectations in the context of use (e.g., increase energy level, social interactions and confidence, reduction in anxiety and irritability), but have seemingly no effect in the long-run. This is supported by our findings showing that a large proportion of student’s population voice concerns about the overall value of substance use in the long-run, tending to consider the usage with negative effects in their life. This is also the case for non-users who present with clear increased awareness of the perceived negative expected outcomes, both in the short and long-run. The misleading expectations students have about the long-term effects from substances, are skewed by their immediate temporal positive effects which are contingently related with their future use (i.e., have reinforcing functions). Decision in taking substance is also heavily influenced by several contextual forces (peers, perceived expectation for use in third-level education, positive elicit effects, absence of risks or control, etc.). In this regard, problem recognition of the perceived short vs. long term beliefs about consequences, are becoming central determinants in explaining the motivation for students to continuous use illegal substances. Thus, focusing at increasing mindful awareness of these contingencies, primarily in recognizing the real vs. expected effects (beliefs about consequences) from substance use on students desired outcomes, can foster the necessary changes to address successful harm-reduction practices. \| \|  \| Social/professional and identity: \| - Identity= Students reported that drug use increases their social role (confidence and social interaction) - Id= students understand the negative effects of drug use in relation to several areas which mapped onto their social identity (a third-level student) - Id.= students express concerns that one of the reasons never using drugs is the negative consequences this may have in their academic role (professional confidence) - Id= student report that drug use negatively impacts their social role (social identity) - Id= student report reasons for stop using drugs in several social identity roles (academic, 36%; personal physical safety; 33% and academic roles; 36%) - Id= students express that their social identity (being a student) substantially increase the risk of drug use, compared to the general population (81.7% more likely for students to use drugs than the general population) - Id = students reported a self-conscious intention (plan) to change their substance use if they find ways to spend time with different social groups than users (group/ social identity) - Id= 39% of students taking drugs reported conflict between their drug use and concerns about their professional identity - Id.= student report using drugs in specific groups, mostly with college, mixed friends or groups. - Id= students report taking substances for their own use by someone who know him/her well and feel they belong in the same group (80%). \| Students also presented the demarcation of substance use effects have on their social role. Apart from increasing confidence and social interaction- both reported as potentially positive effects from substance use- they reported only negative effects on their social roles in different contexts (e.g., academic disruptions, risks in physical safety, reductions in popularity levels, etc.). Notably, this perceived reflections, were found for both non-users, being in the contemplation stage, and users. They both agreed that their social role (being a student) increases the risk for using substances, in comparison to the general population. However, this risk is substantially reduced, when students find ways to spend time with different groups than the one, defined by the substance use. An increase awareness of the latent discrepancy between substance use behaviours and how they threat their social identity, may enhance the target behavior to occur. \| |
| **Step 4: Paradigm Coding** | **Example:** Representation of the narrative statements (clusters of drug-use behaviours), coded around the central phenomena (the COM-B components) and the categories (TDF) | 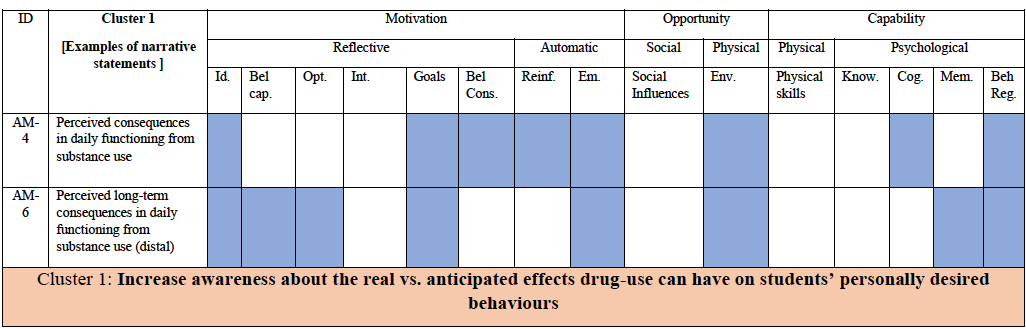  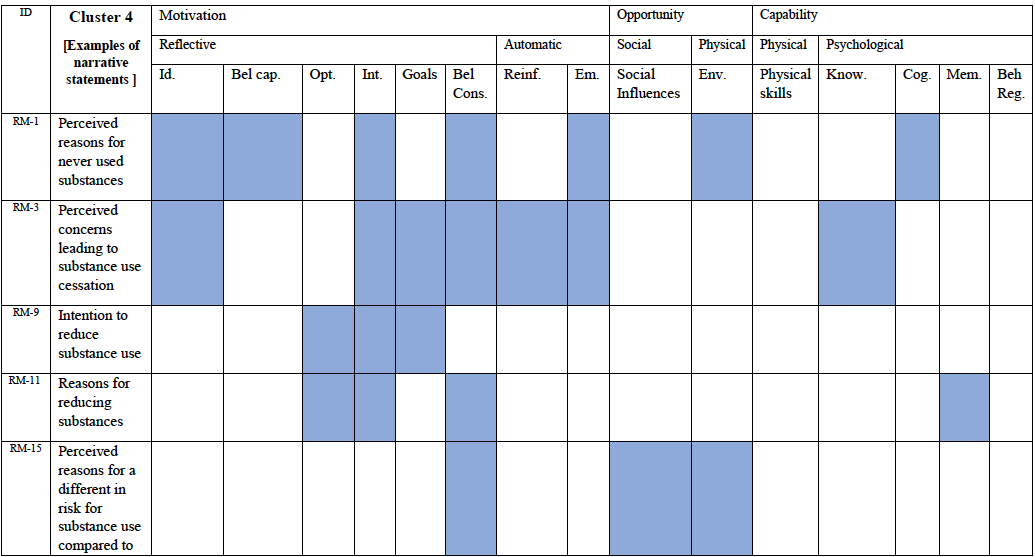  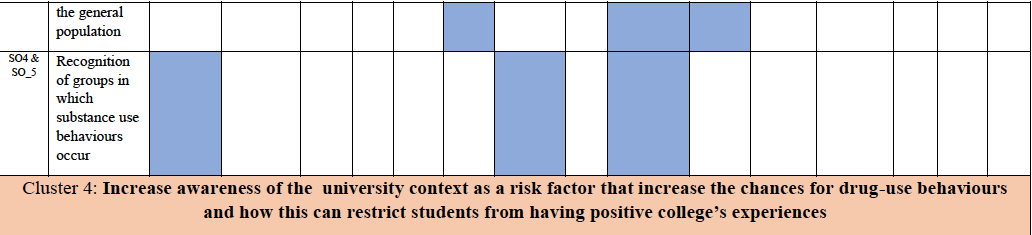 |

Note: we followed the Strauss & Corbin (49) four step procedure to analyze the data in a systematic way .

Supplementary Table A4: Use of the APEASE criteria to identify the BCTs for the MiUSE intervention

| **Cluster of behavior** | **Selected Intervention Function** | **BCTTv1 (Short- list of BCTs)** | |
| --- | --- | --- | --- |
|  |  | **Short List of BCTs** | **Does the BCT meet the APEASE criteria (affordability, practicability, effectiveness/cost-effectiveness, acceptability, side effects/safety, equity) in the context of cluster of behavior to target?** |
| **Cluster 1:** Increase awareness about the short and long-term consequences of substance use in students’ daily functioning. | Education  (selected: 5 BCTs) | - 1. Problem solving | Not applicable for the MiUSE intervention with possible low effectiveness. |
|  |  | 5.1. Information about health consequences | Yes, info about drug use health consequences will be provided |
|  |  | 5.3. Information about social and environmental consequences | Yes, subjunctive norm correction will be part of the intervention. |
|  |  | 5.4. Anticipated regret | Not, this may be practical but possible not acceptable from students- creating an aversive experience we cannot control of it. |
|  |  | 5.6. Information about emotional consequences | Yes, info about the positive effects of valued-living will be provided. |
|  |  | 6.3. Information about others’ approval | Yes, Subjunctive norm correction will be part of the personalized feedback. |
|  |  | 9.2. Pros and cons | Not practical and time consuming to occur within the MiUSE Intervention. |
|  |  | 13.4. Valued self-identity | Yes, this will be part of the value activity and can occur with fair success. |
|  |  | 13.5. Identity associated with changed behaviour | No, possible not acceptable from the targeting group. |
|  | Modelling  (selected: 0 BCTs) | 5.6. Information about emotional consequences | No, possibly not acceptable from the targeting group and not practical to capture modelling for population-based intervention. |
|  |  | 8.1. Behavioral practice/rehearsal | Not applicable and time consuming for this cluster of behaviours to change. |
|  |  | 8.6. Generalization of a target behaviour | Same as above |
|  |  | 13.4. Valued self-identity | Not practical via modelling |
| **Cluster 2:** Promote identification of personally relevant activities (which are they?) which lead to positive expected outcomes in students’ desired behaviours (fun & enjoyment) | Education  (selected: 3 BCTs) | 1.3. Goal setting (outcome) | Yes, this can be achieved via activities promoting value-based actions |
|  |  | 7.2. Cue signalling reward | Yes, providing awareness (knowledge; via plan activities) on how other than drug-activities can also result for students to have fun (the stimulus are the activities) |
|  |  | 8.3. Habit formation | Not applicable and practical within the MiUSE and the cluster of behavior. |
|  |  | 13.2. Framing/reframing | Yes, through the implementation of personally relevant activities the aim is to change students’ attitudes about alternative than drug activities than can lead to positive outcomes (have fun) |
|  | Modelling  (selected: 3 BCTs) | 5.6. Information about emotional consequences | Yes, testimonies of previous MiUSE users can function as models. |
|  |  | 15.1. Verbal persuasion about capability | Yes |
|  |  | 16.3. Vicarious learning | Yes |
|  | Training  (selected: 3 BCTs) | 1.4. Action planning | Yes, detailed planning via committed actions can target this cluster. |
|  |  | 4.1. Instruction on how to perform a behaviour | Not possibly acceptable- students may feel that are being patronized. |
| **Cluster 3:** Increase perceived competence & optimism that an implementation plan of alternative to substance use activities can induce positive expected experiences (fun & enjoyment) | Modelling  (selected: 0 BCTs) | 3.2. Social support (practical) | Not possibly acceptable from students. |
|  |  | 5.6. Information about emotional consequences | Same as above |
|  |  | 6.2. Social comparisons | No, any comparison may not well perceived by students, risking engagement to the intervention |
|  |  | 8.2. Behavioural substitution | No, this would not be accepted from students via role model, risking users’ engagement with the intervention. |
|  |  | 8.4. Habit reversal | Same as above |
|  |  | 8.5. Overcorrection | Not practical and with low rate of success |
|  |  | 8.6. Generalization of a target behaviour | Same as above |
|  |  | 9.1. Credible source | Not acceptable; who is a credible source for students? Difficult to generalize this. |
|  |  | 9.3. Comparative imaging of future outcomes | Not practical |
|  |  | 10.10. Reward outcome | Not applicable |
|  |  | 13.2. Framing/reframing | Not practical and highly risky to be seen as paternalistic when delivered via modelling |
|  | Education  (selected: 7 BCTs) | 1.2. Problem solving | Not practical |
|  |  | 1.3. Goal setting (outcome) | Yes, increase knowledge on how goal setting- other than drugs- can induce positive experiences is relevant to enhancing this cluster of behavior. |
|  |  | 1.8. Behavioural contract | No, it would be possibly perceived too paternalistic. |
|  |  | 1.9. Commitment | Yes, increase knowledge on how commitment to alternative to substance use activities can induce positive experiences can lead to enhancing this cluster of behavior. |
|  |  | 2.2. Feedback on behaviour | Not practical. |
|  |  | 3.3. Social support (emotional) | No, possibly contradictory, if applied- students may support drugs use activities. |
|  |  | 5.4. Monitoring of emotional consequences | Not practical and with low rates of effectiveness- may induce the opposite behaviours. |
|  |  | 5.6. Information about emotional consequences | No, possibly with low value |
|  |  | 6.3. Information about others’ approval | Yes, providing knowledge how alternative to substance use activities would increase others’ approval, would enhance this cluster |
|  |  | 8.1. Behavior practice/rehearsal | Not applicable |
|  |  | 8.2. Behavior substitutions | Yes, provide direct knowledge for substituting substance use activities and their effects, could increase optimism |
|  |  | 8.6. Generalization of a target behaviour | Yes, advice to perform alternatives (value behaviours) in other contexts to build new behavioural repertoires |
|  |  | 10.10. Reward (outcome) | Not practical |
|  |  | 13.1. Identification of a self as a role model | No, possible not acceptable and unaffordable |
|  |  | 13.2. Framing/reframing | Yes, if the framing is linked with imposing positive emotions that can have long-lasting effects as a result of pursuing valued-behaviours. |
|  |  | 15.1. Verbal persuasion about capability | Yes, increase knowledge on how to dispute counterproductive or self-intimidating thoughts, could enhance this cluster. |
|  |  | 15.2. Mental rehearsal of successful performance | Not affordable and practical |
|  |  | 15.4. Self-talk | Not possibly acceptable from students. |
|  |  | 16.2 Imaginary reward | Not practical |
|  |  | 16.3. Vicarious consequences | Not practical |
| **Cluster 4:** Increase awareness regarding the degree to which the university context heightens substance use related behaviours and how these behaviours restrict students from having positive experiences at universities | Education  (selected: 2 BCTs) | 1.6. Discrepancy between current behavior and goal | Not practical and possibly not acceptable from students. |
|  |  | 2.3. Self-monitoring of behaviour | Yes, provide knowledge in relation to the role of the university context in shaping students’ behaviours. |
|  |  | 2.4. Self-monitoring of outcome of behaviours | Not practical- mostly the outcomes can be short-term positive, reinforcing the opposite of the targeting behavior. |
|  |  | 2.5. Monitoring outcome (s) of behaviour by others without feedback | Not practical and risky for reinforcing the unwanted behavior |
|  |  | 4.2. Information about antecedents | Yes, increase knowledge of the university as a context. |
|  |  | 10.11. Future punishment (include threat) | Not practical and possible with low effectiveness. |
|  | Modelling  (selected: 2 BCTs) | 1.6. Discrepancy between current behavior and goal | Not practical and with low rate of effectiveness |
|  |  | 5.6. Information about emotional consequences | Not practical via modelling |
|  |  | 7.1. Prompt/cues | Yes, this will be provided via a video or photos presenting possible influences of behaviours. |
|  |  | 15.4. Self-talk | Yes, showing a model to repeat whether the context heightens substance use decision making. |
| **Cluster 5:** Cultivate mindful awareness of the perceived reasons for using and increase insight as to whether the use leads to desired outcomes in goal-directed behaviours at a long-run | Education  (selected: 6 BCTs) | 1.2. Problem solving | Not practical- too time consuming and probable with low success. |
|  |  | 1.6. Discrepancy between current behaviours and goals | Yes, pointing out the discrepancies would be an excellent BCTs |
|  |  | 2.4. Self-monitoring of the outcomes of behaviours | Yes, highlighting the long-term consequences would enhance this cluster. |
|  |  | 4.1. Instructions on how to perform a behaviour | Yes, increasing knowledge on how to become mindful aware, would enhance this cluster |
|  |  | 4.2. Information about antecedents | Yes, provide information about antecedents (social contextual, internal) through an ABC approach, would predict this cluster of behaviour to change. |
|  |  | 8.1. Behavioural practice/rehearsal | Yes, Prompts practice of mindfulness awareness in different context would assist cultivation of this cluster of behaviour. |
|  |  | 13.4. Valued self-identity | Yes, this can be part of values assessment. |
|  | Modelling  (selected: 5 BCTs) | 4.1. Instructions on how to perform a behavior | Yes, instructions how to become more mindful aware via a video or images, could enhance this cluster of behaviour. |
|  |  | 6.1. Demonstration of the behaviour (modelling) | Yes, as 4.1. |
|  |  | 8.1. Behavioral practice/rehearsal | Yes, prompting users to follow a modelling role in different context could assist the development of this cluster of behaviour. |
|  |  | 8.6. Generalization of a target behavior | Yes, as 8.1. and only if this is encouraged by a statement as part of the skill development. |
|  |  | 15.1. Verbal persuasion about capability | Yes, telling a person that can successfully increase awareness (e.g., testimony). |
|  | Persuasion  (selected: 4 BCTs) | 1.9. Commitment | Yes, if persuasion targeting stimulating actions |
|  |  | 5.2. Salience of consequences | Yes, use any persuasive method increase mindful awareness for the reasons of use and how this impacting the long-term goal, can cultivate changes in this cluster. |
|  |  | 5.6. Information about emotional consequences | Yes, communicate how the change of this behaviour will promote positive long-term outcomes in valued behaviours, can promote this cluster of behavior. |
|  |  | 8.1. Behavioral practice/rehearsal | Not practical via persuasion. |
|  |  | 8.6. Generalization of a target behaviour | No, as above |
|  |  | 13.2. Framing/reframing | Yes, persuasion will be used to enhance how the adoption of mindful decision making can facilitate long-term goals in valued behaviours. |
|  |  | 16.2. Imaginary reward | Not practical via persuasion. |
|  | Training  (selected: 2 BCTs) | 6.1. Demonstration of the behavior | Yes, providing steps on how to cultivate mindful attention would be valuable for enhancing this cluster of behaviour. |
|  |  | 8.7. Graded tasks | Yes, separating the new skill in smaller steps would promote learning of the new skill. |
| **Cluster 6:** Resolve students’ misleading expectations about the expected outcomes from substance use in students’ desired behaviours | Education  (selected: 2 BCTs) | 1.2. Problem solving | Not practical and affordable- too time consuming for a digital intervention |
|  |  | 5.2. Salience of consequences | Yes, providing knowledge that highlight users’ consequences of their behaviours, would support this cluster of behaviour. |
|  |  | 13.2. Framing/reframing | Yes, increasing understanding of behavioural responding (e.g., suggesting awareness of decision making) can enhance understanding on possible misleading expectations about positive misleading effects from substances. |
|  |  | 13.3 Incompatible beliefs | No, too risky to reinforce the unwanted behaviour |
|  |  | 13.4. Valued self-identity | Same as above |
|  | Modelling  (selected: 0 BCTs) | 5.2. Salience of consequences | Not practical and affordable. Also, it may not be acceptable from students. |
|  | Persuasion  (selected: 1 BCTs) | 5.5. Anticipated regret | Yes, if the communication enhances understanding of the possible negative long-term effects in students’ desired behaviours (e.g., less fun from college’s years) from the continuous substance use |
| **Cluster 7:** Increase procedural knowledge on how harm-reduction practices can be implemented within the university context | Education  (selected: 4 BCTs) | 1.8. Behavioural contract | Yes, when students will select their own harm-reduction plan |
|  |  | 4.1. Instructions on how to perform a behavior | Yes, via visual-text based stimuli which will illustrate how harm-reduction can be implemented |
|  |  | 8.6. Generalization of the target behaviour | Yes, increase knowledge on how harm-reduction practices can be implemented in non-academic environments (e.g., parties, festivals, etc.) |
|  |  | 8.7. Graded tasks | Not practical and affordable, given that there will be several harm-reduction practices |
|  |  | 12.3. Avoidance/ reducing exposure to cues for the behaviour | Yes, for those with willingness to change- perhaps as part of the personalized feedback |
|  |  | 16.2. Imaginary reward | Not practical and with questionable effectiveness. |
|  | Modelling  (selected: 2 BCTs) | 1.9. Commitment | Not practical and possible not acceptable- may be perceived as too paternalistic/ didactic |
|  |  | 4.1. Instruction on how to perform a behavior | Yes, if models provide clear instructs on how to perform a harm-reduction practice (images) |
|  |  | 6.1. Demonstration of the behavior | Not practical- too time consuming |
|  |  | 6.3. Information about others approval | Not practical |
|  |  | 8.3. Habit formation | No, possibly not acceptable from students |
|  |  | 9.1. Credible source | No, same as above |
|  | Training  (selected: 2 BCTs) | 1.8. Behavioural contract | Not relevant |
|  |  | 4.1. Instruction on how to perform a behavior | Yes, as part of providing examples, illustrating how harm-reduction practices can be achieved |
|  |  | 6.1. Demonstration of the behavior | Yes |
|  |  | 8.3. Habit formation | Not relevant. |
|  | Incentivisation  (selected: 2 BCTs) | 3.2. Social support (practical) | Not affordable and practical |
|  |  | 3.3. Social support (emotional) | As above |
|  |  | 10.7. Self-incentive | No, possibly not acceptable from students |
|  |  | 10.9. Self-reward | Same as above |
| **Cluster 8:** Promote behavioural awareness and behavioural regulation on whether decision for taking substance use occurs under the influence of peers | Education  (selected: 8 BCTs) | 1.6. Discrepancy between current behaviour and goal | Yes, increase understanding on how users decide to take drug (behavioural regulation) based on what other do (peers’ influence) can enhance this cluster |
|  |  | 4.1. Instruction on how to perform a behaviour | Yes, provide knowledge on how to build this skill (increase awareness and regulation) can |
|  |  | 4.2. Information about antecedents | Yes, if the knowledge focuses at increasing insight of the context as risky environment |
|  |  | 5.2. Salience of consequences | Yes, if knowledge focuses at the behavioural awareness part of this cluster |
|  |  | 8.2. Behavioural substitution | Yes, promote substitution of mindless decision making to mindful (i.e., how my decision is purely mine) can enhance this cluster |
|  |  | 8.7. Graded tasks | Not practical and relevant as the new behavioural skill is not complex |
|  |  | 13.2. Framing/reframing | Yes, if knowledge focuses at enhancing reframing of the benefit of practicing behavioural awareness |
|  |  | 13.4 Valued self-identity | Not practical and possibly not acceptable from students denying changing this behavior. |
|  |  | 15.1. Verbal persuasion about capability | No, possibly not acceptable as students may feel being patronized (what to do with their friend). |
|  |  | 15.2. Mental rehearsal of successful performance | Yes, if this will be integrated as part of the mindful practices skill to be built |
|  |  | 15.4. Self-talk | Not effective and with low spill over implications |
|  | Modelling  (selected: 2 BCTs) | 4.1. Instruction on how to perform a behaviour | Yes, if the new skill includes images presenting others implementing the skill |
|  |  | 6.1. Demonstration of the behaviour | Yes, as above (mix both) |
|  |  | 8.2. Behavioural substitution | Not practical and risky- students may feel patronized |
|  | Training  (selected: 2 BCTs) | 1.8. Behavioural contract | Not practical |
|  |  | 1.9. Commitment | Not practical and possibly low effective |
|  |  | 4.1. Instruction on how to perform a behaviour | Not practical and possibly not acceptable- students may feel being lectured on how to behave |
|  |  | 6.1. Demonstration of the behaviour | Not practical for this function |

Note: Highlighted BCTs with orange represent the selected BCTs for the MyUSE and the ones with grey represent the ones which failed to meet the APEASE criteria.
